# Schisandrin alleviates the cognitive impairment in rats with Alzheimer’s disease by altering the gut microbiota composition to modulate the levels of endogenous metabolites in the plasma, brain, and feces

**DOI:** 10.3389/fphar.2022.888726

**Published:** 2022-09-12

**Authors:** Chengqin Zhang, Ying Zhang, Tiantian Zhao, Tingting Mou, Wang Jing, Jian Chen, Wenqian Hao, Shuo Gu, Meirong Cui, Yue Sun, Binbin Wei

**Affiliations:** Pharmacy Teaching Experimental Center, School of Pharmacy, China Medical University, Shenyang, China

**Keywords:** Alzheimer’s disease, schisandrin, gut microbiota, metabolomics, 16S rRNA gene sequencing

## Abstract

Schisandrin is one of the main active compounds isolated from the fruit of *Schisandrae chinensis* Fructus, which is scientifically proven to have beneficial effects on Alzheimer’s disease (AD) treatment at the cellular and whole organism level. However, the oral availability of schisandrin is very low, thus implying that the underlying mechanism of therapeutic effect on AD treatment is yet to be clarified fully. Therefore, we speculated that the therapeutic effect of schisandrin on AD is mainly by regulating the imbalance of the gut microbiota (GM). In this study, behavioral experiments and H&E staining were used to confirm the pharmacological effects of schisandrin on rats with AD. 16S rDNA gene sequencing and feces, plasma, and brain metabolomics techniques were utilized to investigate the therapeutic effects and the underlying mechanisms of schisandrin on cognitive impairment in rats with AD. The results indicated that schisandrin improved cognitive impairment and hippocampal cell loss in rats. The UPLC-QTOF/MS-based metabolomics studies of the feces, plasma, and brain revealed that 44, 96, and 40 potential biomarkers, respectively, were involved in the treatment mechanism of schisandrin. Schisandrin improved the metabolic imbalance in rats with AD, and the metabolic changes mainly affected the primary bile acid biosynthesis, sphingolipid metabolism, glycerophospholipid metabolism, and unsaturated fatty acid biosynthesis. Schisandrin can improve the GM structure disorder and increase the abundance of beneficial bacteria in the gut of rats with AD. The predictive metagenomics analysis indicated that the altered GM was mainly involved in lipid metabolism, steroid hormone biosynthesis, arachidonic acid metabolism, biosynthesis of unsaturated fatty acids, and bacterial invasion of epithelial cells. Spearman’s correlation analysis showed a significant correlation between affected bacteria and metabolites in various metabolic pathways. Overall, the data underline that schisandrin improves the cognitive impairment in rats with AD by affecting the composition of the GM community, thus suggesting the potential therapeutic effect of schisandrin on the brain–gut axis in rats with AD at the metabolic level.

## 1 Introduction

Alzheimer’s disease (AD), being a neurodegenerative disorder, brought a heavy mental burden and economic pressure to the people worldwide since its onset (Lee et al., 2018). The most compelling hypothesis about the pathophysiology of AD is the accumulation of extracellular amyloid-β (Aβ) plaques and the formation of neurofibrillary hyperphosphorylated tau (p-tau) tangles, eventually leading toward the loss of neurons and synapses (Weiner et al., 2017).

The gut microbiota (GM) is considered as an important companion to help balance the vital life functions of the host through participation in the processes of maintaining the host’s defense and inflammatory responses, digestion, and nutrient bioavailability (Cryan et al., 2020; Pereira et al., 2020). Previous studies suggest that the microbiota and its induced immunity are involved in the pathogenesis and progression of neurodegenerative diseases (Bell et al., 2019; Kundu et al., 2019). Also, there is a clear correlation between the changes in GM and the development of AD. For example, in patients with AD, the abundance of microbiota has changed (Li et al., 2019), and further studies have revealed that the GM may participate in Aβ aggregation (Marizzoni et al., 2020). Therefore, GM can be used as a potential new target for AD treatment, and the pathogenesis of AD and its possible treatment methods can be discovered by studying the GM, which will be of great significance.

Schisandrin is one of the main active ingredients of the *Schisandrae chinensis* Fructus, popular for its therapeutic effect on AD at the molecular, cellular, and whole organism level (Wei et al., 2018; Zhao et al., 2019). Previous studies conducted on the efficacy of schisandrin had a main focus on the brain, and further research has not been conducted on the overall biological activity of schisandrin and its specific treatment mechanisms. The oral bioavailability of schisandrin is only 7.69%, and the blood–brain barrier absorption is 0.1, thereby hindering its absorption by the blood through oral administration. Herein, we speculated that schisandrin may show its biological activity in the intestines, primarily by affecting the genetically modified environment rather than absorption through blood. Therefore, the systematic study about the effects of schisandrin on the brain–gut axis in rats with AD can further clarify the underlying therapeutic mechanisms of treatment and would be of great significance in evaluating the effect of GM on metabolism.

Metabolomics can systematically analyze small molecules in cells, tissues, organs, or biological systems which helps out in understanding the mechanism of changes being occurred (Alseekh and Fernie 2018). In this regard, the 16S rDNA gene can accurately quantify all the bacterial species in the GM, as it is the most commonly used molecular marker for the systematic classification of prokaryotic microorganisms (Lu et al., 2020). The expression of the core functions of GM can alter with the change of the gut microenvironment, which would have variable effects on many tissues of the host. Metabolomics analysis helps in comparing the metabolic changes in the GM with the metabolic changes in the host (Evans et al., 2014). Considering the complex mode of action of schisandrin, this study mainly uses metabolomics combined with 16S rDNA gene sequencing technology to systematically explore its protective effect on rats with AD for the first time at the level of brain–gut axis. Furthermore, database prediction and correlation statistics methods were also employed in the research process, which provides research directions for future in-depth research.

## 2 Materials and methods

### 2.1 Chemical reagents

Methanol, formic acid, and acetonitrile were obtained from Merck AG (Germany), and the water used throughout the experiment was sourced from Watsons. Among other reagents, D-(+) Galactose was purchased from Beijing Soleibao Technology Co., Ltd. (China), Aβ_25-35_ was purchased from Shanghai Yuanye Biological Technology Co., Ltd. (China), and schisandrin (purity >98%) was purchased from the National Institute for Food and Drug Control. The chemical structure for schisandrin is provided as a diagram in Supplementary Figure S1.

### 2.2 Animals

Regarding the animals utilized in this study, male SD rats (8 weeks old, 200–250 g) were purchased from Changsheng Biotechnology Co., Ltd. (China), from them 18 rats were housed in the cages (one cage for every three rats) which were placed in a 23 ± 2°C and 50 ± 5% humidity environment (food and water were freely available). The animal study was carried out in accordance with the guidelines for Animal Experimentation of China Medical University. The Animal Ethics Committee had approved the institutional protocol (CMU2019257). The Morris water maze (MWM) test was carried out in an air-regulated and soundproofed experimental room, and the rats got used to the experimental room at least 1 h beforehand.

### 2.3 Animal experiment design

Aβ_25-35_ peptide solution was incubated in sterile water at 37°C for 7 days before injection. A total of 18 rats were randomly divided (Excel’s RAND function) into three groups (blank group, model group, and schisandrin administration group (Sch group), *n* = 6). From day 1 after the completion of adaptive feeding, the rats from the model and Sch groups were administered with D-galactose (120 mg kg^−1^) through intraperitoneal injection and were given AlCl_3_ (80 mg kg^−1^) by oral gavage, whereas the blank group rats were given an equal volume of normal saline at the same time, and this whole administration was administered once a day until the MWM test was conducted. From the sixth week onward, rats from the Sch group were treated with schisandrin at the rate of 10 mg kg^−1^, calculated according to the conversion algorithm of human and rat body surface area. The rats from the model and blank groups received an equal volume of normal saline. This administration continued until the ninth week. On the last day of the eighth week, all the rats were kept fasted for 24 h before the surgery. On day 1 of the ninth week, all the rats were anaesthetized with 10% chloral hydrate and were fixed on the brain stereotaxic instruments. According to the stereotactic map of the brain, 3 μL of Aβ_25-35_ (1 mg ml^−1^) was injected into each bilateral hippocampal CA1 area of rats from the model and Sch groups, whereas, the rats in the blank group were injected with an equal volume of saline (the anterior fontanelle is the zero starting point. The rats were drilled 3.6 mm behind the anterior fontanelle and 2.6 mm on the left and right sides of the median raphe by the stereotaxic apparatus. With a depth of 2.9 mm and a slow injection of 3 μL Aβ_25-35,_ the rat scalp tissues were sutured with sterile surgical sutures). The rats from the Sch group were continuously given schisandrin for 1 week after the cerebroventricular injection test.

### 2.4 Morris water maze test

After the drug treatment, the rats were trained on the MWM, in which the main body of the water maze device was a circular pool (diameter, 150 cm; height, 60 cm), and it was divided into four quadrants artificially. The escape platform was located at the first quadrant (1 cm below the water surface). From day 1–5, the rats under observation were randomly placed into the water maze with their backs facing toward the pool wall. The recording time was set to 60 s, during which the rats climb to the platform and stays there for 3 s, after which the time recording stopped and started counting as the escape latency (if the platform was not found for more than 60 s, the rats were guided to see the venue and allowed to stay on the platform for 10 s, the escape latency was counted as 60 s). The rats from all the groups were trained daily in four quadrants. The platform was removed from the first quadrant on day 4 to conduct the space exploration experiment. The number of times was recorded for each rat crossing the target platform position, and that number was considered as final statistics which the rat has made crossings in the first quadrant in 60 s. The experimental arrangement plan is provided in Supplementary Figure S2.

### 2.5 Sample collection and processing

#### 2.5.1 Sample collection

The samples were collected after the MWM tests were over. Fresh feces samples were collected in sterile EP tubes directly from the anus, and were stored at −80°C for metabolomics analysis and 16S rDNA gene sequencing. On the next day, the rats were anesthetized by intraperitoneal injection of 10% chloral hydrate (0.25 mg 100 g^−1^), and plasma samples were collected from the abdominal aorta into EP tubes coated with sodium heparin. The centrifuged plasma supernatant was stored at −80°C (centrifuged at 1,600 × g for 10 min at 4°C) for metabolomics analysis. Thereafter, the rats were sacrificed by decapitation, and their brains were immediately removed. One half of each brain was soaked in 4% paraformaldehyde solution, and the other half was stored at −80°C for Ultra Performance Liquid Chromatography/Quadrupole time-of-flight mass spectrometer (UPLC-QTOF/MS)-based analysis.

#### 2.5.2 Sample processing

Each 200 µl aliquot of the plasma sample was added with 600 µL of cooled methanol. The mixtures were then vortexed (60 s) and placed at −20°C (20 min) to allow the proteins to precipitate. Thereafter, the mixtures were centrifuged (6,200 × g, 10 min, 4°C), and all the supernatants transferred to EP tubes were allowed to evaporate until dryness with the stream of nitrogen (35°C). Thereafter, 200 µl of 80% methanol solution was added to the dried residue. After the mixture was completely dissolved, it was centrifuged (6,200 ×g, 10 min, 4°C), and the supernatant was taken for analysis.

Regarding the feces sample, each 50 mg feces sample was mixed with 1,500 µl cooled methanol, and the mixture was homogenized (60 HZ, 30 s) and vortexed (60 s) to allow the proteins to precipitate. Then, the mixture was placed at −20°C (20 min) and centrifuged twice (6,200 ×g, 10 min, 4°C) and all the supernatants were filtered through a 0.22 µm microporous millipore membrane. Thereafter, it was centrifuged (6,200 ×g, 10 min, 4°C), and the supernatant was taken for analysis.

For the brain samples, each 50 mg brain samples was mixed with 1,000 µL of 50% cooled methanol solution, and the mixture was homogenized (35 HZ, 60 s, 4°C) and subsequently vortexed (60 s). Then, the sample was placed at −20 °C (20 min) to allow the proteins to precipitate. The mixtures were then centrifuged (6,200 ×g, 10 min, 4°C), and all the supernatants transferred to EP tubes were allowed to evaporate until dryness with the stream of nitrogen (35°C). After this, 200 µL of 80% methanol solution was added to the dried residue, and the mixture was completely dissolved and centrifuged (6,200 ×g, 10 min, 4°C), and the supernatant was taken for analysis.

When all the samples were processed separately, quality control (QC) samples with a volume of 120 µl were configured at the same time. The QC sample was a mixture of all samples.

### 2.6 H&E staining

For the H&E staining process, the rats’ brain was extracted from 4% paraformaldehyde solution and embedded in paraffin. Coronal sections of 4 μm thick were prepared for staining, and following steps were performed: section dewaxing, hematoxylin stain (5 min), water rinsing (30 min) for blue return, eosin staining for 2 min, dehydrating, and sealing. After that, each rat was randomly selected in four parts to take a photograph (the magnification of visualized damaged brain area was 20 times). The neurons were quantified in five randomly selected hippocampal CA1 micrographs from each segment, and the number of CA1 cells was counted. Only the cells with apparent nuclei and nucleoli were measured, while the other cells without prominent nuclei and nucleoli were excluded from the quantitative range.

### 2.7 UPLC-QTOF/MS-based metabolomics

Regarding the metabolomics analysis, metabolic profiling was analyzed on the UPLC-QTOF/MS system (Waters Corporation, Milford, MA, United States) in the presence of positive (ES+) and negative ionization sources (ES-) mode (Zhou et al., 2019). Water ACQUITY UPLC H-Class/Xevo used for UPLC analysis. For this purpose, 3 µL samples were injected into the Acquity HSS (High class) T3 column (2.1 mm × 100 mm, 1.8 μm, Waters). During this procedure, the UPLC analysis conditions were as follows: column temperature, 35°C; flow rate, 0.4 ml min^−1^; and sample manager temperature, 4°C. For the metabolomics analysis of feces, the prevailing conditions were as: gradient system (ES+): methanol (A) and a mixture of 0.1% formic acid in water (B) (0–2 min, A, 5%; 2–3 min, A, 5–20%; 3–16, A, 20–80%; 16–17 min, A, 80–95%; 17–20 min, A, 95%*;* 20–20.1 min, A, 95–5%; 20.1–23 min, A, 5%); The gradient system (ES-): acetonitrile (A) and water (B) (0–2 min, A, 5%; 2–3 min, A, 5–20%; 3–16 min, A, 20–80%; 16–17 min, A, 80–95%; 17–20 min, A, 95%; 20–20.1 min, A, 95%–5%; and 20.1–23 min, A, 5%).

The parametric conditions for the brain metabolomics analysis were as follows: the gradient system (ES+): acetonitrile (A) and a mixture of 0.1% formic acid in water (B) (0–2 min, A, 2%; 2–6 min, A, 2–40%; 6–18 min, A, 40–98%; 18–21 min, A, 98%; 21–21.1 min, A, 98–2%; 21.1–23 min, A, 2%); and the gradient system (ES-): acetonitrile (A) and water (B) (0–2 min, A, 2%; 2–6 min, A, 2–60%; 6–14 min, A, 60–98%; 14–17 min, A, 98%; 17–17.1 min, A, 98–2%; 17.1–20 min, A, 2%).The conditions in the plasma metabolomics analysis were as follows: the gradient system (ES+): acetonitrile (A) and a mixture of 0.1% formic acid in water (B) (0–2 min, A, 5%; 2–18 min, A, 5–95%; 18–21 min, A, 95%; 21–21.1 min, A, 95–5%; 21.1–23 min, A, 5%); and the gradient system (ES-): acetonitrile (A) and water (B) (0–2 min, A, 5%; 2–18 min, A, 5–95%; 18–21 min, A, 95%; 18–20 min, A, 5%; 21–21.1 min, A, 95–5%; 21.1–23 min, A, 5%).

Similarly, the MS parameters were as follows: nebulizing gas, high-purity nitrogen (N_2_); collision gas, ultra-high pure helium (He); desolvent gas flow, 800 (L^−1^Hr); source temperature, 120°C; desolvent temperature, 500°C; conical air, 50 L^−1^ h; collision energy, 20–45 v; capillary voltage: plasma sample, 2.0 kV; brain sample, 2.5 kV; feces sample, 1.5 kV; and scanning range, m/z 100–1,200 Da. Leucine keorphin (200 pg μL^−1^) has a mass of 556.2771 in ES+ and 554.2615 in ES-. At the start of sequencing, we ran 10 QC samples and inserted a QC sample between every six sequences to further ensure the stability of the instrument.

### 2.8.16S rDNA gene sequencing analysis

The total DNA of feces samples was extracted using E.Z.N.A. ^®^ Stool DNA Kit. The V3–V4 region of the 16S rDNA gene was amplified by PCR. The bacterial primers were 341F (5′-CCTACGGGNGGCWGCAG-3′) and 805R (5′-GACTACHVGGGTATTCTAATCC-3′). Thereafter, the PCR products were purified (AMPure XT beads, Beckman Coulter Genomics, Danvers, MA, United States) and quantified (Qubit, Invitrogen, United States). Agilent 2100 Bioanalyzer (Agilent, United States) and Library Quantification Kit for Illumina (Kapa Biosciences, Woburn, MA, United States) were employed to evaluate the size and quantity of the amplicon library, respectively. The NovaSeq PE250 platform was used for sequencing. Quality filtering was performed according to fqtrim (v0.94). Vsearch software (v2.3.4) was used to filter the chimeric sequences.

### 2.9 Data analysis

MarkerLynx within MassLynx software version 4.1 (Waters Corp. Milford, United States) was employed to process all the collected UPLC-QTOF/MS data. Progenesis QI V 2.3 software (Waters, Milford, MA, United States) was used for peak detection and alignment, and EZinfo 3.0 software was used for the multivariate data analysis. To match and distinguish Rt-m/z pairs of different metabolites being selected in each group, an online database named HMDB (Human Metabolome Database, http://www.hmdb.ca/) was consulted and high-precision ion fragments were utilized. Enrichment analysis of the metabolomics pathways was performed through online websites, such as https://www.metaboanalyst.ca/and https://www.kegg.jp/, and the HMDB number of the identified differential metabolites were entered into the Metaboanalyst online data analysis software. Fisher’s exact test was selected as the enrichment method, and the rat *Rattus norvegicus* (rat) (KEGG) library was selected as the database for pathway analysis.

The data collected from the MWM and H&E staining were expressed as the mean ± standard deviation (SD), and the statistical significance was measured by GraphPad Prism 8.0.1. Spearman’s correlation analysis of the GM and endogenous metabolites was performed by the OmicStudio tools at https://www.omicstudio.cn/tool.

## 3 Results

### 3.1 Morris water maze test

**FIGURE 1:**
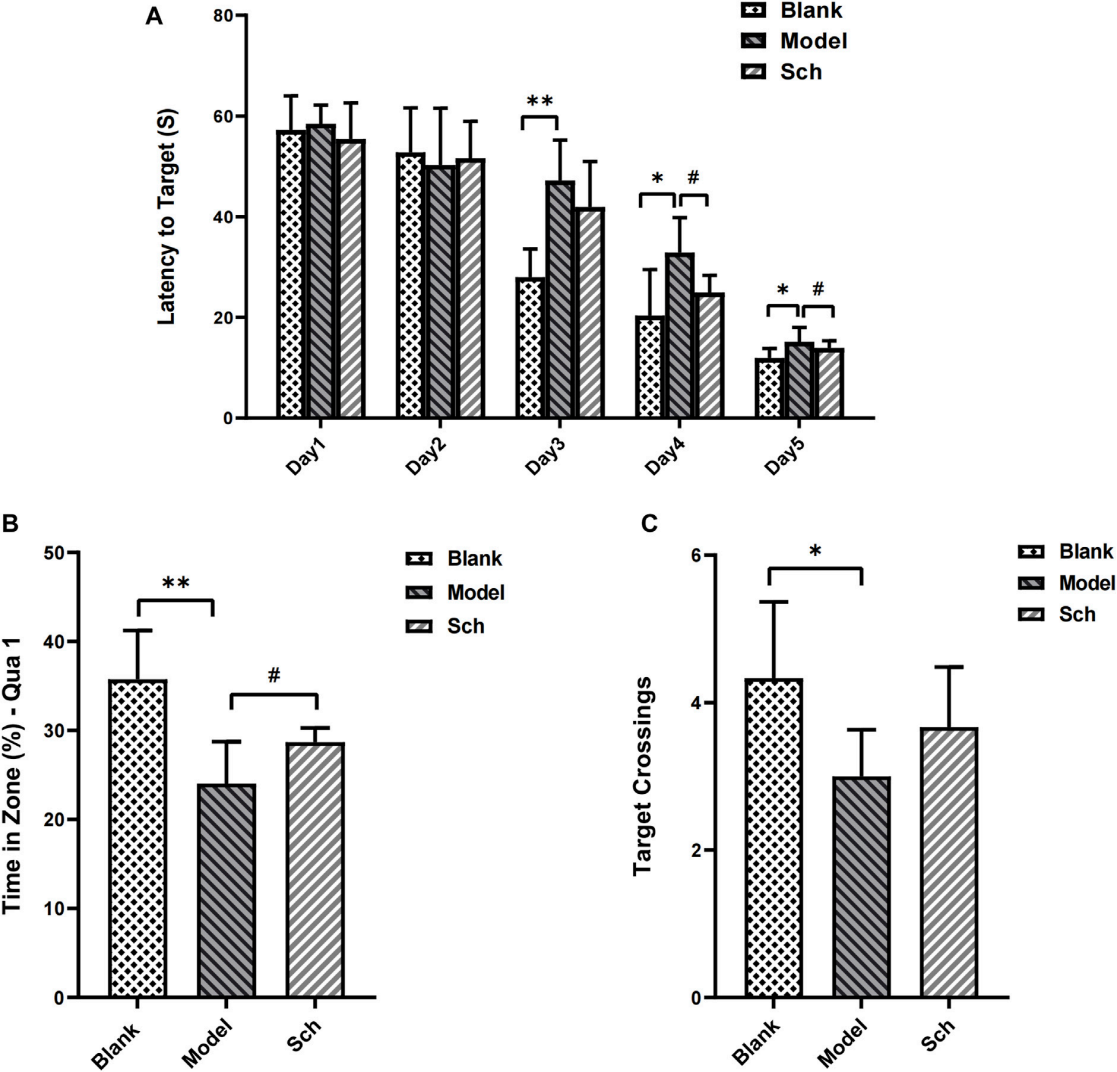
Effects of schisandrin on the cognitive impairment of rats with AD in the MWM test. **(A)** Escape latency of rats in the space exploration experiment; **(B)** time percentage of the rats stayed in the target quadrant in spatial exploration test; and **(C)** number of times the rats crossed the platform in the spatial exploration test. (*p ≤ 0.05, **p ≤ 0.01 vs. blank group; #*p* < 0.05 vs. model group). All results are expressed as the mean ± SD (*n* = 6).

The escape latency of each group gradually shortened with the increase in training time. From the third day, the rats in the model group took longer than for those in the blank group to find an escape platform (Figure 1A), indicating that the AD model was successfully established. Similarly, the rats from the Sch group showed a significant effect on the fourth day of training as well. In the space exploration test, residence time for the rats from the model group in the target quadrant (Figure 1B), and the number of target platform crossings (Figure 1C) were significantly reduced as compared to the blank and Sch groups. These results indicated that the rats in the blank and Sch groups had spent more time and stayed longer in the first quadrant, indicating better spatial memorability.

### 3.2 H&E staining

**FIGURE 2:**
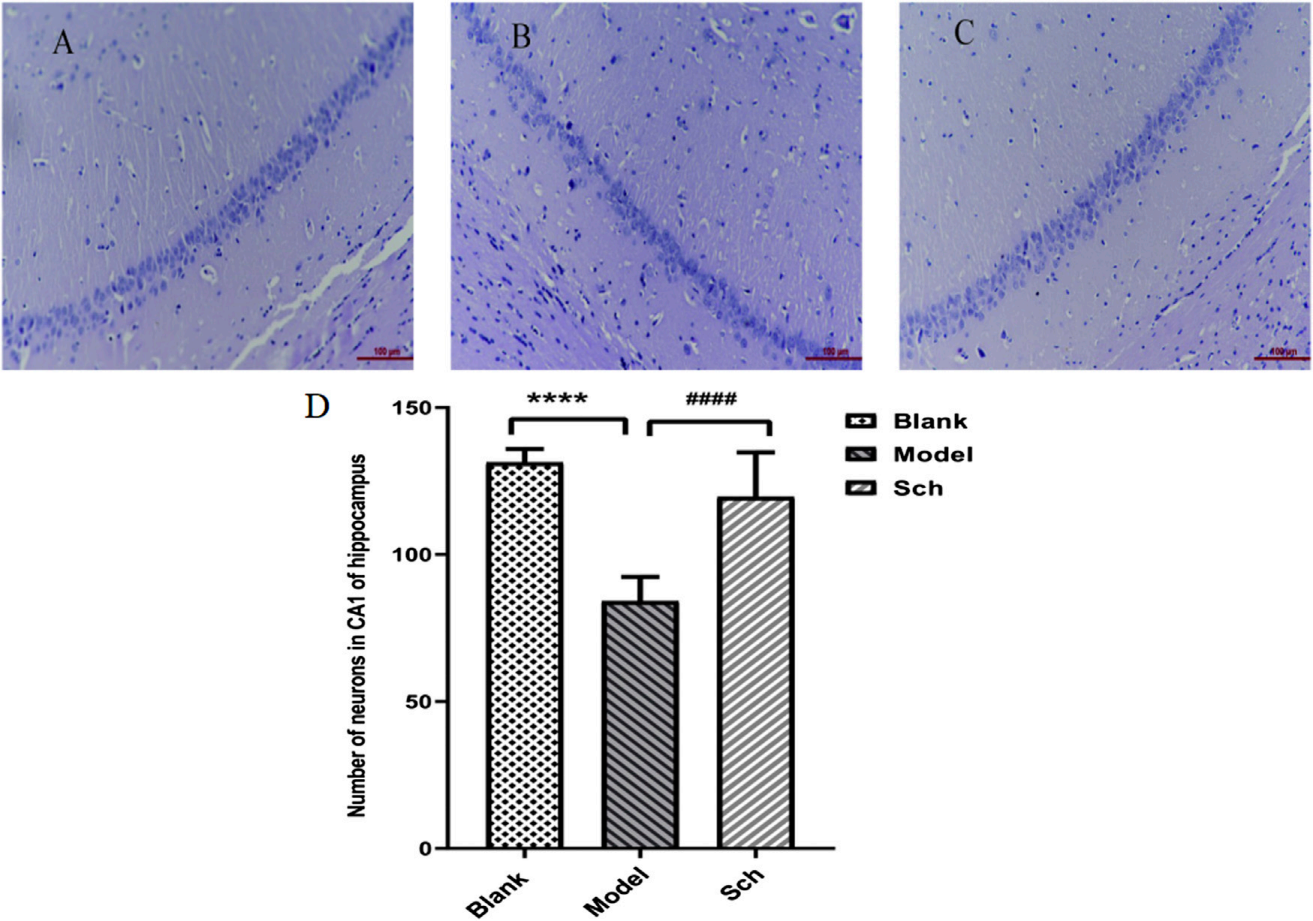
H&E staining. CA1 pyramidal cell layer in the hippocampus: **(A)** blank group; **(B)** model group; **(C)** Sch group; and **(D)** number of neurons in the hippocampus (****p ≤ 0.0001 vs. blank group; ####*p* ≤ 0.0001 vs. model group). All results are expressed as the mean ± SD (*n* = 6).

The CA1 pyramidal cell layer of the Sch group was observed to be thicker than that of the model group (Figures 2A–C). The number of neurons in the CA1 of the hippocampus was significantly more in the Sch group than the model group, indicating that schisandrin had a protective effect on the loss of hippocampal neurons in the diseased rats (Figure 2D, *p* < 0.0001).

### 3.7 Metabolomics research

#### 3.7.1 UPLC-QTOF/MS results

**FIGURE 3:**
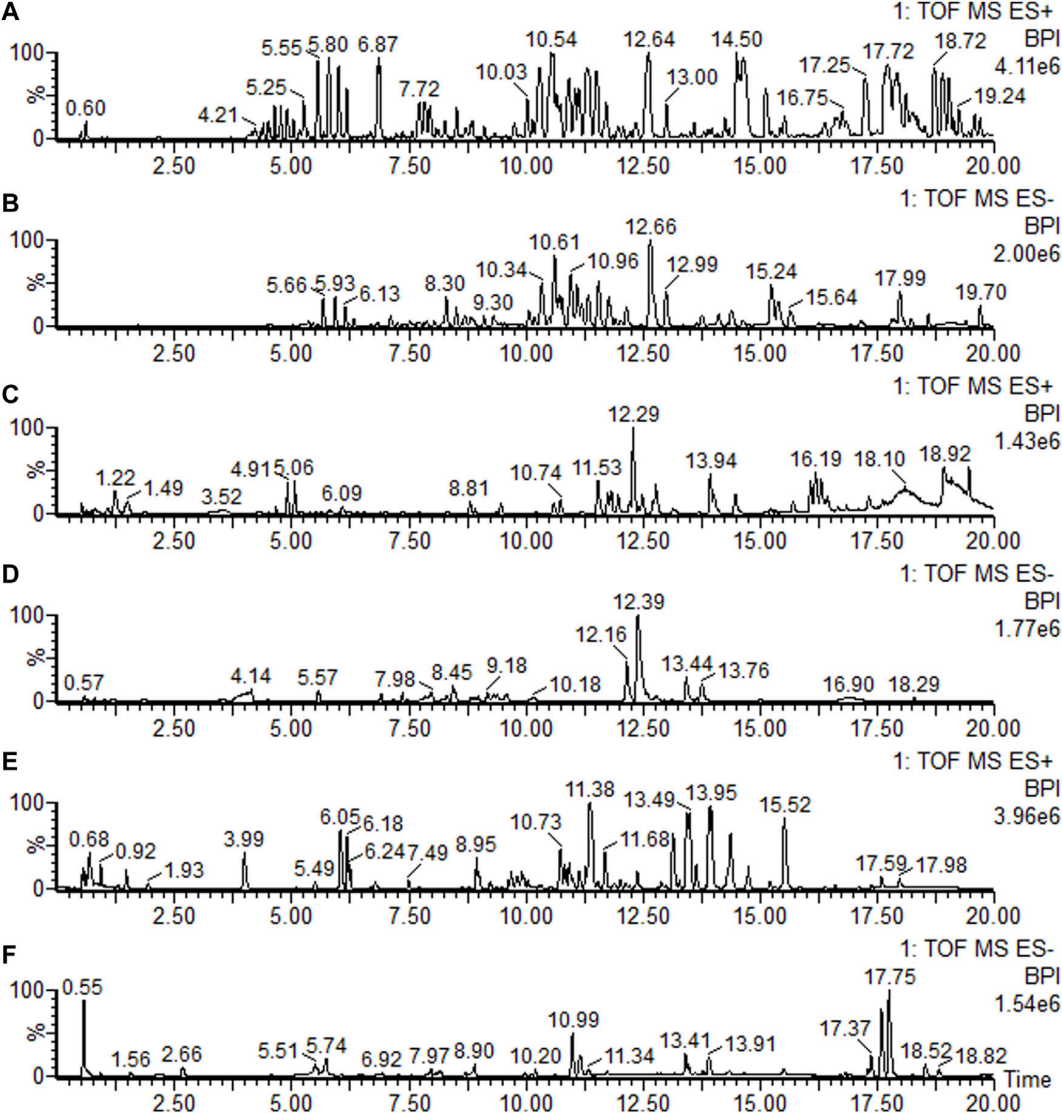
Base peak ion current (BPI) chromatograms of the feces, brain, and plasma in positive mode (ES+) and negative mode (ES-), respectively. **(A)** Feces sample in positive mode; **(B)** feces sample in negative mode; **(C)** brain sample in positive mode; **(D)** brain sample in negative mode; **(E)** plasma sample in positive mode; and **(F)** plasma sample in negative mode.

Under the elution program, the base peak images (BPI) showed ideal separation results. The BPIs of the plasma, feces, and brain samples are shown in ES+ and ES- modes, respectively, in Figures 3A-F.

#### 3.7.2 Identification of potential differential metabolites

**FIGURE 4:**
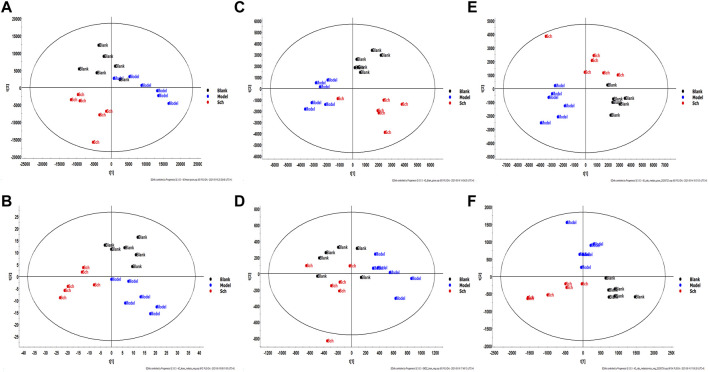
Multivariate statistical analysis figure of PLS-DA: **(A)** feces sample in positive mode; **(B)** feces sample in negative mode; **(C)** brain sample in positive mode; **(D)** brain sample in negative mode; **(E)** plasma sample in positive mode; and **(F)** plasma sample in negative mode.

**FIGURE 5:**
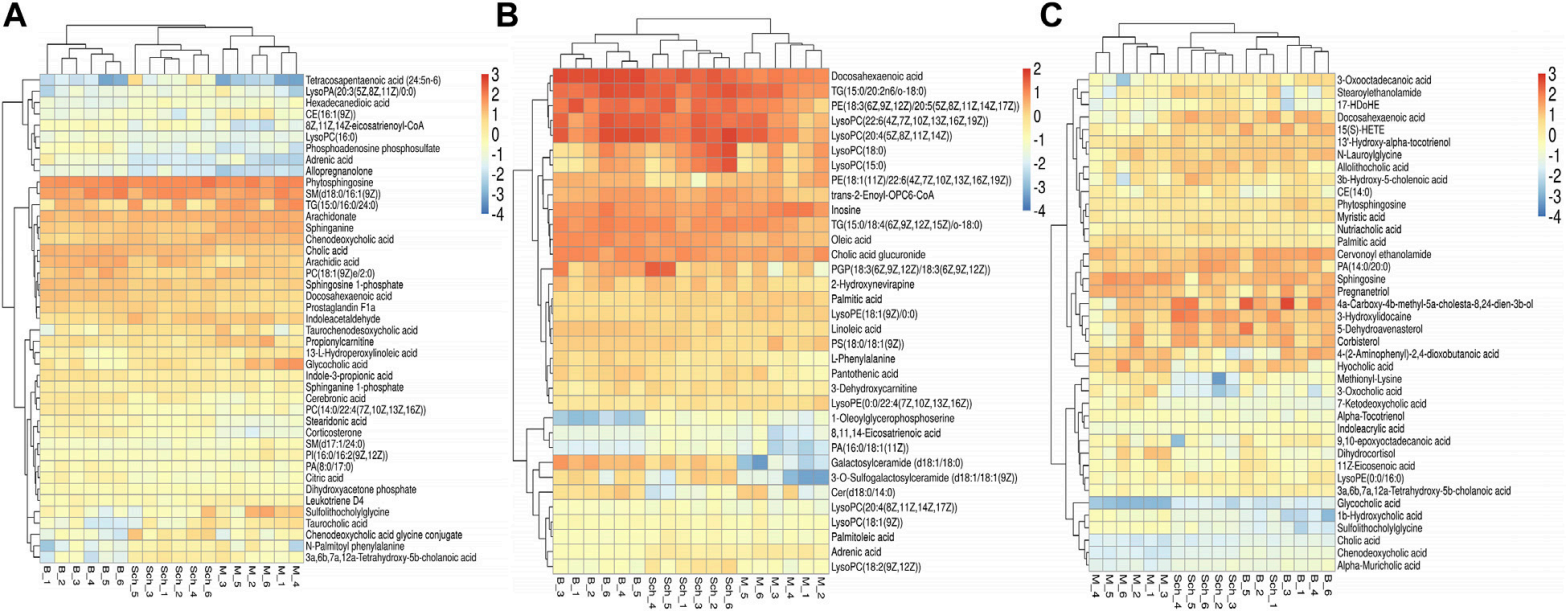
Heatmap cluster analysis of the plasma, brain, and feces. **(A)** plasma sample; **(B)** brain sample; and **(C)** feces sample.

The results from the PLS-DA (Partial least square-discriminant analysis) (Figures 4A-F) showed that the model and blank groups have a clear cluster classification, thus suggesting that there was a certain difference in metabolism between the model and blank group rats. According to OPLS-DA (Orthogonal partial least square-discriminant analysis), marked the compounds with VIP (Variable importance in the projection) > 1 on the S-plot, for which the OPLS-DA figures and S-plot are provided in the supplementary materials (Supplementary Figure S3). Furthermore, 44, 96, and 40 potential biomarkers (Mass error <5, CV (Cross-validated residuals) ≤ 30 (in QC), VIP >1, P (*p* value) < 0.05) were found in the feces, plasma, and brain samples, respectively, and are listed in Tables 1–3. The metabolites with significant changes in the metabolic level were analyzed by heatmap clustering, which showed that the metabolite levels in the Sch group have different degrees of callback as compared to the model group (Figures 5A-C).

**TABLE 1:** Potential biomarkers found in the brain.

Compound	HMDB ID	Adducts	Formula	FC	*p*	Description	Pathways
13.39_255.2336m/z	HMDB0000220	M-H	C16H32O2	1.46	0.010	Palmitic acid	Biosynthesis of unsaturated fatty acids
13.72_281.2494m/z	HMDB0000207	M-H	C18H34O2	1.91	0.000	Oleic acid	Biosynthesis of unsaturated fatty acids
12.74_279.2336m/z	HMDB0000673	M-H	C18H32O2	2.03	0.007	Linoleic acid	Biosynthesis of unsaturated fatty acids
12.38_303.2338m/z	HMDB0060102	M-H	C20H32O2	1.88	0.000	Arachidonate	Biosynthesis of unsaturated fatty acids
13.10_305.2491m/z	HMDB0002925	M-H	C20H34O2	3.12	0.000	Dihomo-gamma-linolenic acid	Biosynthesis of unsaturated fatty acids
12.12_327.2340m/z	HMDB0002183	M-H	C22H32O2	1.85	0.000	Docosahexaenoic acid	Biosynthesis of unsaturated fatty acids
7.51_798.4456m/z	HMDB0009137	M + K	C43H70NO8P	7.81	0.000	PE (18:3 (6Z,9Z,12Z)/20:5 (5Z,8Z,11Z,14Z,17Z))	Glycerophospholipid metabolism
17.91_781.5655n	HMDB0007889	M + H, M + Na	C44H80NO8P	3.31	0.000	PC(14:0/22:4 (7Z,10Z,13Z,16Z))	Glycerophospholipid metabolism
12.37_495.3329n	HMDB0010382	M + H, M + Na, M + H- H_2_O	C24H50NO7P	5.94	0.000	LysoPC(16:0)	Glycerophospholipid metabolism
13.71_834.5265m/z	HMDB0009045	M + FA-H	C45H76NO8P	2.32	0.019	PE (18:1 (11Z)/22:6 (4Z,7Z,10Z,13Z,16Z,19Z))	Glycerophospholipid metabolism
9.22_521.3463n	HMDB0002815	M + Cl, M + FA-H	C26H52NO7P	1.53	0.001	LysoPC(18:1 (9Z))	Glycerophospholipid metabolism
12.13_655.4721m/z	HMDB0007858	M- H_2_O -H	C37H71O8P	4.97	0.000	PA (16:0/18:1 (11Z))	Glycerophospholipid metabolism
18.28_789.5487n	HMDB0010163	M-H, M + FA-H	C42H80NO10P	2.05	0.001	PS(18:0/18:1 (9Z))	Glycerophospholipid metabolism
17.25_512.5055m/z	HMDB0011759	M + H	C32H65NO3	34.44	0.000	Cer(d18:0/14:0)	Sphingolipid metabolism
20.75_727.5985n	HMDB0010709	M + H- H_2_O, M + Na	C42H81NO8	60.46	0.001	Galactosylceramide (d18:1/18:0)	Sphingolipid metabolism
7.49_828.5261m/z	HMDB0012317	M + Na	C42H79NO11S	59.85	0.001	3-O-sulfogalactosylceramide (d18:1/18:1 (9Z))	Sphingolipid metabolism
5.28_996.2765m/z	HMDB0011121	M + H- H_2_O	C37H58N7O18P3S	3.37	0.000	trans-2-Enoyl-OPC6-CoA	alpha-Linolenic acid metabolism
4.48_166.0856m/z	HMDB0000159	M + H	C9H11NO2	5.29	0.000	L-phenylalanine	Phenylalanine, tyrosine, and tryptophan biosynthesis
4.76_219.1117n	HMDB0000210	M + H, M + Na, M + H- H_2_O	C9H17NO5	5.18	0.000	Pantothenic acid	Pantothenate and CoA biosynthesis
2.12_305.1000m/z	HMDB0013912	M + Na	C15H14N4O2	3.65	0.000	2-Hydroxynevirapine	Drug metabolism - cytochrome P450
4.05_267.0741m/z	HMDB0000195	M-H	C10H12N4O5	1.56	0.003	Inosine	Purine metabolism
5.14_826.7426n	HMDB0043684	M + H, M + NH_4_	C54H98O5	3.97	0.000	TG (15:0/18:4 (6Z,9Z,12Z,15Z)/o-18:0)	
5.90_1072.6588n	HMDB0116850	M + H- H_2_O, M + K	C53H102O17P2	5.04	0.000	CL (8:0/8:0/10:0/i-18:0)	
11.88_568.3406m/z	HMDB0010404	M + H	C30H50NO7P	5.85	0.000	LysoPC(22:6 (4Z,7Z,10Z,13Z,16Z,19Z))	
5.27_876.8398m/z	HMDB0043481	M + NH_4_	C56H106O5	4.02	0.000	TG (15:0/20:2n6/o-18:0)	
13.61_331.2652m/z	HMDB0002226	M-H	C22H36O2	2.22	0.000	Adrenic acid	
8.31_522.2838m/z	HMDB0061694	M-H	C24H46NO9P	18.85	0.000	1-Oleoylglycerophosphoserine	
8.50_588.3301m/z	HMDB0010396	M + FA-H	C28H50NO7P	1.72	0.015	LysoPC(20:4 (8Z,11Z,14Z,17Z))	
12.05_253.2177m/z	HMDB0003229	M-H	C16H30O2	1.56	0.015	Palmitoleic acid	
14.13_523.3639n	HMDB0010384	M + H, M + Na	C26H54NO7P	9.38	0.002	LysoPC(18:0)	
14.04_481.3174n	HMDB0010381	M + H, M + Na, M + H- H_2_O	C23H48NO7P	12.55	0.007	LysoPC(15:0)	
0.63_184.0741m/z	HMDB0006831	M + K	C7H15NO2	2.58	0.001	3-Dehydroxycarnitine	
13.07_530.3254m/z	HMDB0011493	M + H	C27H48NO7P	11.68	0.006	LysoPE (0:0/22:4 (7Z,10Z,13Z,16Z))	
9.15_478.2951m/z	HMDB0011506	M-H	C23H46NO7P	1.26	0.010	LysoPE (18:1 (9Z)/0:0)	
11.89_544.3405m/z	HMDB0010395	M + H	C28H50NO7P	5.49	0.000	LysoPC(20:4 (5Z,8Z,11Z,14Z))	
11.83_520.3421m/z	HMDB0010386	M + H	C26H50NO7P	4.08	0.000	LysoPC(18:2 (9Z,12Z))	
20.47_828.5593m/z	HMDB0009778	M + NH_4_	C41H79O13P	53.62	0.002	PI(16:0/16:0)	
21.12_796.5489m/z	HMDB0012382	M + H- H_2_O	C44H80NO10P	27.79	0.003	PS(18:0/20:3 (8Z,11Z,14Z))	
6.44_829.4421m/z	HMDB0013568	M + H-H2O	C42H72O13P2	317.00	0.000	PGP (18:3 (6Z,9Z,12Z)/18:3 (6Z,9Z,12Z))	
7.80_619.2890m/z	HMDB0002577	M + Cl	C30H48O11	1.67	0.002	Cholic acid glucuronide	

**TABLE 2:** Potential biomarkers found in the feces.

Compound	HMDB ID	Adducts	Formula	FC	*p*	Description	Pathways
16.22_321.2419m/z	HMDB0003876	M + H	C20H32O3	7.45	0.035	15(S)-HETE	Arachidonic acid metabolism
12.88_300.2899m/z	HMDB0000252	M + H	C18H37NO2	3.94	0.032	Sphingosine	Sphingolipid metabolism
11.54_318.3011m/z	HMDB0004610	M + H	C18H39NO3	39.13	0.022	Phytosphingosine	Sphingolipid metabolism
17.48_411.3621m/z	HMDB0006852	M + H	C29H46O	4.70	0.021	5-Dehydroavenasterol	Steroid biosynthesis
17.40_443.3506m/z	HMDB0001181	M + H	C29H46O3	15.55	0.049	4a-Carboxy-4b-methyl-5a-cholesta-8,24-dien-3b-ol	Steroid biosynthesis
4.24_162.0548m/z	HMDB0004077	M + H	C9H7NO2	79.71	0.034	4,6-Dihydroxyquinoline	Tryptophan metabolism
4.18_208.0603m/z	HMDB0000978	M + H	C10H9NO4	11.78	0.019	4-(2-Aminophenyl)-2,4-dioxobutanoic acid	Tryptophan metabolism
12.28_392.2924n	HMDB0000518		C24H40O4	Infinity	0.000	Chenodeoxycholic acid	Primary bile acid biosynthesis
10.19_408.2865n	HMDB0000619		C24H40O5	769.99	0.000	Cholic acid	Primary bile acid biosynthesis
7.96_465.3085n	HMDB0000138		C26H43NO6	4832.63	0.017	Glycocholic acid	Primary bile acid biosynthesis
18.53_255.2319m/z	HMDB0000220	M-H	C16H32O2	2.78	0.025	Palmitic acid	Biosynthesis of unsaturated fatty acids
9.21_329.2482m/z	HMDB0002183	M + H	C22H32O2	4.42	0.029	Docosahexaenoic acid	Biosynthesis of unsaturated fatty acids
9.28_814.5521m/z	HMDB0009610	M + Cl	C45H82NO7P	73.90	0.001	PE (22:4 (7Z,10Z,13Z,16Z)/P-18:0)	Glycerophospholipid metabolism
16.12_508.3761m/z	HMDB0013122	M + H	C26H54NO6P	40.32	0.041	LysoPC(P-18:0)	Glycerophospholipid metabolism
7.15_424.2821n	HMDB0000399	M-H, 2M-H	C24H40O6	5.60	0.047	3a,6b,7a,12a-Tetrahydroxy-5b-Cholanoic acid	Fructose and mannose metabolism
4.43_365.2311m/z	HMDB0003259	M + H	C21H32O5	17.20	0.013	Dihydrocortisol	Steroid hormone biosynthesis
17.07_227.2007m/z	HMDB0000806	M-H	C14H28O2	2.56	0.032	Myristic acid	Fatty acid biosynthesis
4.27_251.1752m/z	HMDB0060655	M + H	C14H22N2O2	10.99	0.043	3-Hydroxylidocaine	Drug metabolism - cytochrome P450
3.06_188.0701m/z	HMDB0000734	M + H	C11H9NO2	363.97	0.037	Indoleacrylic acid	
16.78_337.2738m/z	HMDB0006070	M + H	C21H36O3	5.59	0.037	Pregnanetriol	
10.43_373.2749m/z	HMDB0013627	M + H	C24H36O3	4.24	0.030	Cervonoyl ethanolamide	
6.83_424.2818n	HMDB0000307	M-H, 2M-H	C24H40O6	26.98	0.017	1b-Hydroxycholic acid	
15.89_297.2433m/z	HMDB0010736	M-H	C18H34O3	4.91	0.020	3-Oxooctadecanoic acid	
9.25_408.2866n	HMDB0000760	M-H, M + Cl, 2M-H	C24H40O5	7.59	0.013	Hyocholic acid	
9.15_406.2710n	HMDB0000502	M-H, 2M-H, M + Cl	C24H38O5	94.59	0.001	3-Oxocholic acid	
13.55_453.2857n	HMDB0011473	M-H, 2M-H	C21H44NO7P	3.41	0.003	LysoPE (0:0/16:0)	
6.96_512.2683m/z	HMDB0002639	M-H	C26H43NO7S	29.56	0.000	Sulfolithocholylglycine	
7.91_553.2847m/z	HMDB0028978	2M-H	C11H23N3O3S	34.17	0.003	Methionyl-Lysine	
19.78_309.2793m/z	HMDB0002231	M-H	C20H38O2	3.72	0.042	11Z-eicosenoic acid	
10.26_328.3219m/z	HMDB0013078	M + H	C20H41NO2	11.61	0.011	Stearoylethanolamide	
10.41_390.2766n	HMDB0000467	M-H, 2M-H	C24H38O4	4.80	0.000	Nutriacholic acid	
11.12_425.3425m/z	HMDB0006327	M + H	C29H44O2	8.69	0.027	alpha-Tocotrienol	
12.68_441.3363m/z	HMDB0012560	M + H	C29H44O3	15.57	0.001	13′-Hydroxy-alpha-tocotrienol	
14.15_297.2432m/z	HMDB0061650	M-H	C18H34O3	7.49	0.011	9,10-Epoxyoctadecanoic acid	
14.29_376.2975n	HMDB0000381	M-H, 2M-H	C24H40O3	3.96	0.016	Allolithocholic acid	
15.55_374.2820n	HMDB0000308	M-H, 2M-H	C24H38O3	4.55	0.019	3b-Hydroxy-5-cholenoic acid	
15.94_326.3056m/z	HMDB0002088	M + H	C20H39NO2	242.81	0.022	N-oleoylethanolamine	
17.67_411.3625m/z	HMDB0033825	M + H	C29H46O	2.68	0.012	Corbisterol	
18.56_677.5102m/z	HMDB0114783	M + H	C37H73O8P	3.31	0.020	PA (14:0/20:0)	
19.76_631.5246m/z	HMDB0006725	M + Cl	C41H72O2	3.28	0.050	CE (14:0)	
4.73_258.2067m/z	HMDB0013272	M + H	C14H27NO3	2.32	0.027	N-lauroylglycine	
7.44_345.2427m/z	HMDB0010213	M + H	C22H32O3	66.12	0.010	17-HDoHE	
8.34_406.2714n	HMDB0000391	M-H, 2M-H	C24H38O5	9.26	0.042	7-ketodeoxycholic acid	
8.98_408.2867n	HMDB0000506	M-H, 2M-H	C24H40O5	169.04	0.002	alpha-Muricholic acid	

**TABLE 3:** Potential biomarkers found in the plasma.

Compound	HMDB ID	Adducts	Formula	FC	*p*	Description	Pathways
15.31_295.2282m/z	HMDB0004702	M-H	C18H32O3	4.45	0.008	12,13-EpOME	Linoleic acid metabolism
19.43_826.5606m/z	HMDB0007889	M + FA-H	C44H80NO8P	3.85	0.001	PC(14:0/22:4 (7Z,10Z,13Z,16Z))	Linoleic acid metabolism
13.78_312.2299n	HMDB0003871	M-H, M + Na-2H	C18H32O4	3.74	0.001	13-L-hydroperoxylinoleic acid	Linoleic acid metabolism
12.30_379.2491n	HMDB0000277	M + H, M + Na	C18H38NO5P	2.80	0.000	Sphingosine 1-phosphate	Sphingolipid metabolism
12.61_382.2728m/z	HMDB0001383	M + H	C18H40NO5P	1.87	0.012	Sphinganine 1-phosphate	Sphingolipid metabolism
12.09_302.3058m/z	HMDB0000269	M + H	C18H39NO2	5.03	0.000	Sphinganine	Sphingolipid metabolism
18.80_725.5581m/z	HMDB0013464	M + Na	C39H79N2O6P	3.21	0.004	SM(d18:0/16:1 (9Z))	Sphingolipid metabolism
10.81_318.3014m/z	HMDB0004610	M + H	C18H39NO3	4.55	0.000	Phytosphingosine	Sphingolipid metabolism
10.82_424.2826n	HMDB0000399	M- H_2_O -H, M-H	C24H40O6	9.22	0.003	3a,6b,7a,12a-Tetrahydroxy-5b-cholanoic acid	Fructose and mannose metabolism
20.79_168.9903m/z	HMDB0001473	M-H	C3H7O6P	1.23	0.019	Dihydroxyacetone phosphate	Fructose and mannose metabolism
0.60_1029.2710n	HMDB0060376	M-H, M + Na-2H, M + FA-H, M- H_2_O -H	C37H58N7O19P3S	1.36	0.016	3-Oxo-OPC6-CoA	alpha-Linolenic acid metabolism
13.86_277.2171m/z	HMDB0006547	M + H	C18H28O2	3.54	0.024	Stearidonic acid	alpha-Linolenic acid metabolism
18.51_699.5236n	HMDB0008984	M + NH_4_, M + K	C39H74NO7P	1.80	0.006	PE (16:1 (9Z)/P-18:1 (11Z))	Glycerophospholipid metabolism
18.64_790.5401m/z	HMDB0008975	M + Na-2H	C43H80NO8P	3.72	0.000	PE (16:1 (9Z)/22:2 (13Z,16Z))	Glycerophospholipid metabolism
14.69_494.3261m/z	HMDB0010382	M-H	C24H50NO7P	2.39	0.002	LysoPC(16:0)	Glycerophospholipid metabolism
12.94_433.2369m/z	HMDB0007852	M-H	C21H39O7P	2.57	0.000	LysoPA (0:0/18:2 (9Z,12Z))	Glycerophospholipid metabolism
18.43_1055.3646n	HMDB0003947	M + H, M + K	C41H68N7O17P3S	4.39	0.002	8Z,11Z,14Z-eicosatrienoyl-CoA	Biosynthesis of unsaturated fatty acids
13.44_330.3377m/z	HMDB0002212	M + NH4	C20H40O2	6.58	0.000	Arachidic acid	Biosynthesis of unsaturated fatty acids
17.59_305.2480m/z	HMDB0060102	M + H	C20H32O2	2.30	0.000	Arachidonate	Biosynthesis of unsaturated fatty acids
17.37_329.2480m/z	HMDB0002183	M + H	C22H32O2	2.29	0.005	Docosahexaenoic acid	Biosynthesis of unsaturated fatty acids
4.55_209.0448m/z	HMDB0000205	M + FA-H	C9H8O3	1.22	0.018	Phenylpyruvic acid	Phenylalanine metabolism
0.97_164.0481n	HMDB0012225	M + H, M + NH_4_	C9H8O3	4.98	0.000	Enol-phenylpyruvate	Phenylalanine metabolism
20.48_393.2981m/z	HMDB0000518	M + H	C24H40O4	5.07	0.000	Chenodeoxycholic acid	Primary bile acid biosynthesis
11.13_408.2893n	HMDB0000619	M + NH_4_, M + Na, M + K	C24H40O5	3.84	0.023	Cholic acid	Primary bile acid biosynthesis
11.53_449.3157n	HMDB0000637	M + H, M + Na	C26H43NO5	21.71	0.001	Chenodeoxycholic acid glycine conjugate	Primary bile acid biosynthesis
14.22_431.3162m/z	HMDB0012458	M + H	C27H42O4	3.02	0.022	7-alpha-Hydroxy-3-oxo-4-cholestenoate	Primary bile acid biosynthesis
9.93_465.3107n	HMDB0000138	M + H, M + Na, M + K	C26H43NO6	9.92	0.015	Glycocholic acid	Primary bile acid biosynthesis
9.06_515.2942n	HMDB0000036	M + H, M + NH_4_, M + K, M + Na	C26H45NO7S	12.19	0.004	Taurocholic acid	Primary bile acid biosynthesis
8.59_499.2956n	HMDB0000951	M-H, M + Na-2H	C26H45NO6S	0.02	8.589	Taurochenodesoxycholic acid	Primary bile acid biosynthesis
15.50_525.0182m/z	HMDB0001134	M + NH_4_	C10H15N5O13P2S	9.87	0.011	Phosphoadenosine phosphosulfate	Sulfur metabolism
0.56_168.0895n	HMDB0001431	M + H, M + Na	C8H12N2O2	3.54	0.000	Pyridoxamine	Vitamin B6 metabolism
0.57_192.0261n	HMDB0000094	M-H, M + Na-2H	C6H8O7	4.31	0.000	Citric acid	Citrate cycle (TCA cycle)
15.50_311.1613m/z	HMDB0000338	M + Na	C18H24O3	3.30	0.000	2-Hydroxyestradiol	Steroid hormone biosynthesis
9.64_346.2147n	HMDB0001547	M-H, M + FA-H	C21H30O4	7.38	0.001	Corticosterone	Steroid hormone biosynthesis
9.98_363.2546m/z	HMDB0001449	M + FA-H	C21H34O2	279.31	0.016	Allopregnanolone	Steroid hormone biosynthesis
9.96_408.2875n	HMDB0000432	M-H, M + Na-2H, M + FA-H	C24H40O5	3.02	0.033	3a,7b,12a-Trihydroxy-5a-Cholanoic acid	Steroid hormone biosynthesis
18.79_807.5770n	HMDB0007989	M + H, M + NH_4_	C46H82NO8P	3.07	0.000	PC(16:0/22:5 (4Z,7Z,10Z,13Z,16Z))	Arachidonic acid metabolism
6.10_519.2496m/z	HMDB0003080	M + Na	C25H40N2O6S	3.25	0.000	Leukotriene D4	Arachidonic acid metabolism
0.62_201.0373m/z	HMDB0000211	M + Na-2H	C6H12O6	2.32	0.013	myo-Inositol	Phosphatidylinositol signaling system
10.45_552.3299n	HMDB0002513	M-H, M + Na-2H	C30H48O9	462.96	0.009	Lithocholate 3-O-glucuronide	Pentose and glucuronate interconversions
2.30_219.1114n	HMDB0000210	M + H, M + Na	C9H17NO5	4.83	0.000	Pantothenic acid	Pantothenate and CoA biosynthesis
15.83_549.3799n	HMDB0011148	M + H, M + Na	C28H56NO7P	3.54	0.000	PC(18:1 (9Z)e/2:0)	Ether lipid metabolism
8.94_569.3120m/z	HMDB0001097	M + H	C34H40N4O4	3.80	0.014	Protoporphyrinogen IX	Porphyrin and chlorophyll metabolism
13.08_186.9825m/z	HMDB0001553	M + K	C5H8O3S	2.03	0.000	2-Oxo-4-methylthiobutanoic acid	Cysteine and methionine metabolism
9.23_246.2435m/z	HMDB0000806	M + NH_4_	C14H28O2	3.68	0.000	Myristic acid	Fatty acid biosynthesis
7.48_218.2121m/z	HMDB0000638	M + NH_4_	C12H24O2	4.27	0.000	Dodecanoic acid	Fatty acid biosynthesis
0.66_381.0267m/z	HMDB0060418	M + H	C10H13N4O8PS	3.58	0.000	6-Thioxanthine 5′-monophosphate	Drug metabolism - other enzymes
0.60_977.3134n	HMDB0001521	M-H, M + Na-2H, M- H_2_O -H	C35H62N7O17P3S	1.30	0.048	Tetradecanoyl-CoA	Fatty acid elongation
1.44_160.0764m/z	HMDB0001190	M + H	C10H9NO	3.70	0.000	Indoleacetaldehyde	Tryptophan metabolism
4.28_640.6002m/z	HMDB0000658	M + NH_4_	C43H74O2	7.36	0.002	CE (16:1 (9Z))	Steroid biosynthesis
1.06_267.0736m/z	HMDB0000195	M-H	C10H12N4O5	195.24	0.000	Inosine	Purine metabolism
0.60_764.4704m/z	HMDB0112282	M + FA-H	C37H70NO10P	1.39	0.012	PS(15:0/16:1 (9Z))	
0.91_204.1239m/z	HMDB0000201	M + H	C9H17NO4	5.11	0.011	L-acetylcarnitine	
1.26_218.1394m/z	HMDB0000824	M + H	C10H19NO4	6.05	0.008	Propionylcarnitine	
1.86_172.9904m/z	HMDB0015167	M + Na-2H	C5H4N4S	7.09	0.028	Mercaptopurine	
10.09_441.2415m/z	HMDB0114747	M- H_2_O -H	C23H41O7P	8.70	0.017	LysoPA (20:3 (5Z,8Z,11Z)/0:0)	
10.13_377.2699m/z	HMDB0002226	M + FA-H	C22H36O2	52.03	0.001	Adrenic acid	
10.33_373.2753m/z	HMDB0013627	M + H	C24H36O3	3.73	0.027	Cervonoyl ethanolamide	
10.57_943.8130m/z	HMDB0043023	M + K	C58H112O6	5.87	0.042	TG (15:0/16:0/24:0)	
10.84_334.2964m/z	HMDB0011532	M + NH_4_	C18H36O4	5.28	0.000	MG (0:0/15:0/0:0)	
10.98_496.3038n	HMDB0010318	M-H, M + Na-2H	C27H44O8	2.64	0.012	Pregnanediol-3-glucuronide	
11.53_414.3014m/z	HMDB0062332	M + H	C26H39NO3	24.97	0.000	N-docosahexaenoyl GABA	
12.90_493.3173n	HMDB0010383	M + H, M + Na	C24H48NO7P	3.90	0.005	LysoPC(16:1 (9Z)/0:0)	
12.92_522.2846m/z	HMDB0061694	M-H	C24H46NO9P	7.24	0.002	1-Oleoylglycerophosphoserine	
13.04_285.2071m/z	HMDB0000672	M-H	C16H30O4	3.80	0.000	Hexadecanedioic acid	
13.91_476.3612n	HMDB0030271	M + Na, M + K	C28H48N2O4	3.25	0.000	Dehydrocarpaine I	
14.27_478.2941m/z	HMDB0011475	M-H	C23H46NO7P	1.82	0.004	LysoPE (0:0/18:1 (11Z))	
14.28_436.2829m/z	HMDB0011152	M-H	C21H44NO6P	2.43	0.005	PE (P-16:0e/0:0)	
14.35_290.1428m/z	HMDB0015690	M + H	C16H20ClN3	2.55	0.000	Chloropyramine	
14.61_572.3723m/z	HMDB0010401	M + H	C30H54NO7P	3.20	0.003	LysoPC(22:4 (7Z,10Z,13Z,16Z))	
14.69_571.3369m/z	HMDB0115488	M + Na-2H	C28H55O8P	2.38	0.002	PA (8:0/17:0)	
14.70_314.2456n	HMDB0000782	M-H, M + Na-2H	C18H34O4	10.10	0.000	Octadecanedioic acid	
14.76_402.3952m/z	HMDB0039540	M + NH_4_	C24H48O3	7.02	0.000	Cerebronic acid	
14.79_547.3645n	HMDB0010392	M + H, M + Na	C28H54NO7P	3.03	0.000	LysoPC(20:2 (11Z,14Z))	
15.05_536.3729m/z	HMDB0011491	M + H	C27H54NO7P	2.91	0.001	LysoPE (0:0/22:1 (13Z))	
15.29_359.2949m/z	HMDB0006322	M + H	C24H38O2	242.45	0.000	Tetracosapentaenoic acid (24:5n-6)	
17.09_256.2641m/z	HMDB0012273	M + H	C16H33NO	2.20	0.003	Palmitic amide	
17.56_528.4969m/z	HMDB0112110	M + NH_4_	C32H62O4	3.63	0.026	FAHFA (16:0/9-O-16:0)	
17.58_330.2780n	HMDB0011533	M + H, M + Na	C19H38O4	7.36	0.000	MG (0:0/16:0/0:0)	
17.96_314.2461n	HMDB0004704	M + Na, M + K, M + H	C18H34O4	3.53	0.000	9,10-DHOME	
18.50_898.7284m/z	HMDB0008323	M + H	C52H100NO8P	10.28	0.001	PC(20:1 (11Z)/24:1 (15Z))	
18.51_395.2207m/z	HMDB0002685	M + K	C20H36O5	1.99	0.003	Prostaglandin F1a	
18.79_927.5382m/z	HMDB0009792	M + K	C47H85O13P	5.00	0.003	PI(16:0/22:3 (10Z,13Z,16Z))	
19.91_496.5078m/z	HMDB0029801	M + NH_4_	C32H62O2	3.42	0.017	8-Dotriacontenoic acid	
20.43_823.5457m/z	HMDB0010608	M + Na	C44H81O10P	3.32	0.002	PG (18:0/20:3 (5Z,8Z,11Z))	
3.86_187.0642n	HMDB0000734	M + H, M + NH_4_	C11H9NO2	4.79	0.000	Indoleacrylic acid	
4.25_261.1444m/z	HMDB0011170	M + H	C11H20N2O5	3.25	0.011	gamma-Glutamylisoleucine	
6.05_446.2240m/z	HMDB0000851	M + NH_4_	C18H28N4O8	4.36	0.000	Pyridinoline	
7.35_824.6750m/z	HMDB0011695	M + Na	C46H94N2O6P+	4.45	0.001	SM(d17:1/24:0)	
7.35_961.6197m/z	HMDB0061392	M + K	C52H93NO10P+	4.02	0.001	PC(DiMe(11,3)/DiMe(13,5))	
7.36_824.5320m/z	HMDB0009780	M + NH_4_	C41H75O13P	4.55	0.001	PI(16:0/16:2 (9Z,12Z))	
8.23_513.2777n	HMDB0002639	M + H, M + K, M + NH_4_, M + Na	C26H43NO7S	20.42	0.026	Sulfolithocholylglycine	
8.43_190.0871m/z	HMDB0002302	M + H	C11H11NO2	3.93	0.004	Indole-3-propionic acid	
9.53_498.2895m/z	HMDB0000874	M-H	C26H45NO6S	5.82	0.014	Tauroursodeoxycholic acid	
9.73_990.5182m/z	HMDB0116076	M + Na	C45H83N3O15P2	2.93	0.023	CDP-DG (a-13:0/i-20:0)	
9.98_448.3069m/z	HMDB0062339	M + FA-H	C25H41NO3	8.66	0.012	N-palmitoyl phenylalanine	

FC, Max fold change; *p*: *p* value.

#### 3.7.3 Analysis of metabolic pathways in the plasma, brain, and feces

**FIGURE 6:**
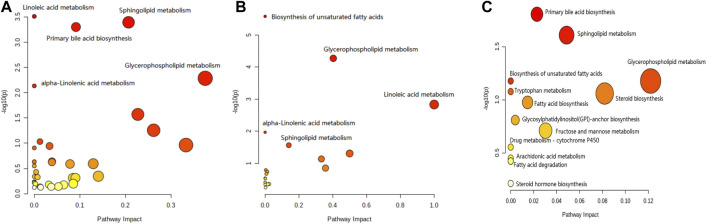
Pathway analysis of the feces metabolomics. **(A)** Plasma sample; **(B)** brain sample; and **(C)** feces sample.

The results for the pathway analysis of the plasma and brain are expressed as bubble plots. A total of five metabolic pathways were enriched in the plasma (*p* ≤ 0.05, Figure 6A), namely, linoleic acid metabolism, sphingolipid metabolism, primary bile acid biosynthesis, glycerophospholipid metabolism, and alpha-linolenic acid metabolism. Similarly, five metabolic pathways were enriched in the brain (*p* ≤ 0.05, Figure 6B), namely, unsaturated fatty acid biosynthesis, glycerophospholipid metabolism, linoleic acid metabolism, alpha-linolenic acid metabolism, and sphingolipid metabolism. According to the results from pathway analysis of feces (Figure 6C), the metabolites were found mainly involved in the primary bile acid biosynthesis, sphingolipid metabolism, glycerophospholipid metabolism, unsaturated fatty acid biosynthesis, fatty acid biosynthesis, tryptophan metabolism, glycosylphosphatidylinositol (GPI)-anchor set biosynthesis, fructose, mannose metabolism, drug metabolism-cytochrome P450, arachidonic acid metabolism, fatty acid degradation, steroid biosynthesis, and steroid hormone biosynthesis.

### 3.3 16S rDNA gene sequencing analysis

#### 3.3.1 Alpha diversity

**FIGURE 7:**
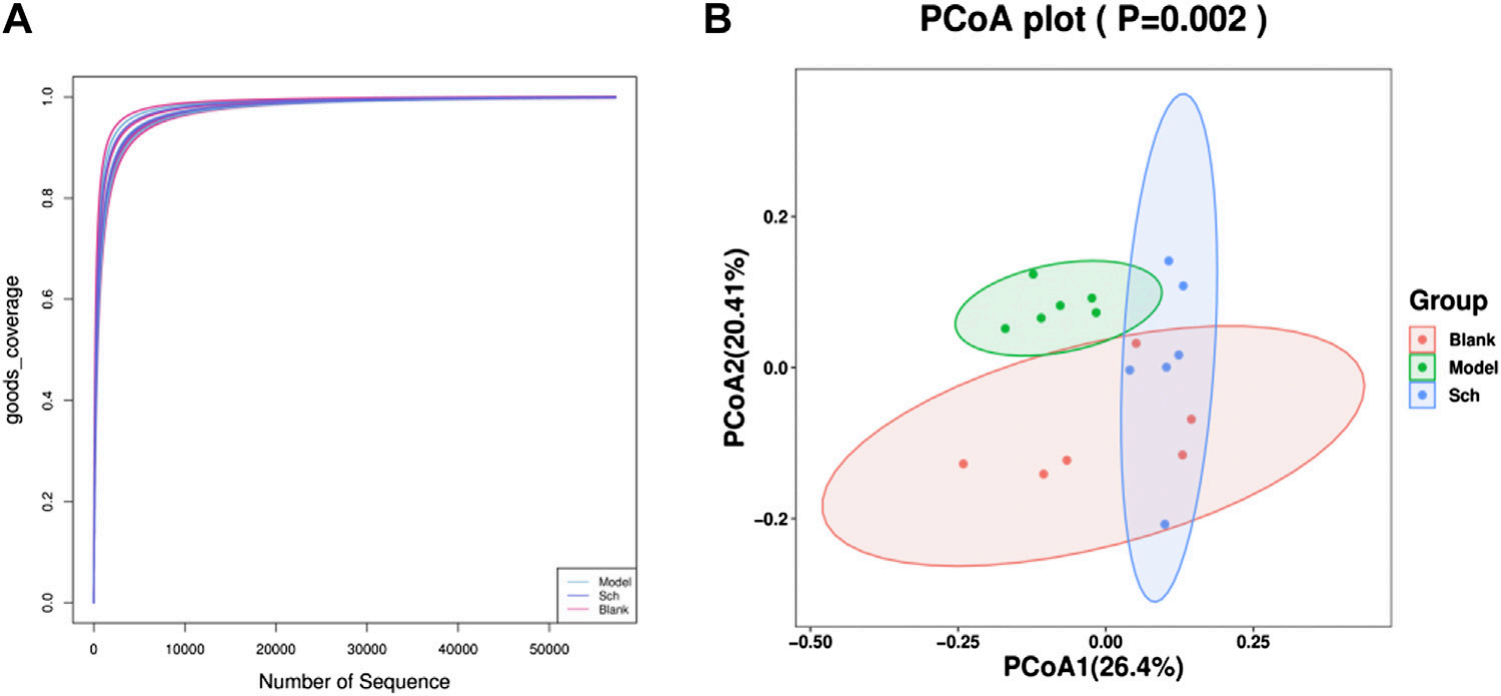
Diversity analysis of the GM. **(A)** Good’s coverage rarefaction curve of alpha diversity analysis; **(B)** PCoA analysis of beta diversity analysis.

Good’s coverage index refers to the microbial coverage, which is an index reflecting whether the sequencing results represent the actual microbial diversity in the sample. In the present study, the slope of the rarefaction curve tended to be 0, indicating that the sequencing was deeply saturated in our samples (Figure 7A).

#### 3.3.2 Beta diversity analysis

The PCoA (Principal coordinate analysis) score chart (Figure 7B) revealed that the microbial community structure composition for the model, blank, and Sch groups had apparent clusters, thereby indicating significant differences in the GM of the three groups. The distance between the blank and Sch groups was close, indicating that the changes in the GM community for the Sch group tended to the level of the blank group, which suggests that schisandrin can promote the GM structure imbalance in rats with AD to approach the GM structure of rats in the blank group.

### 3.4 Species analysis

**FIGURE 8:**
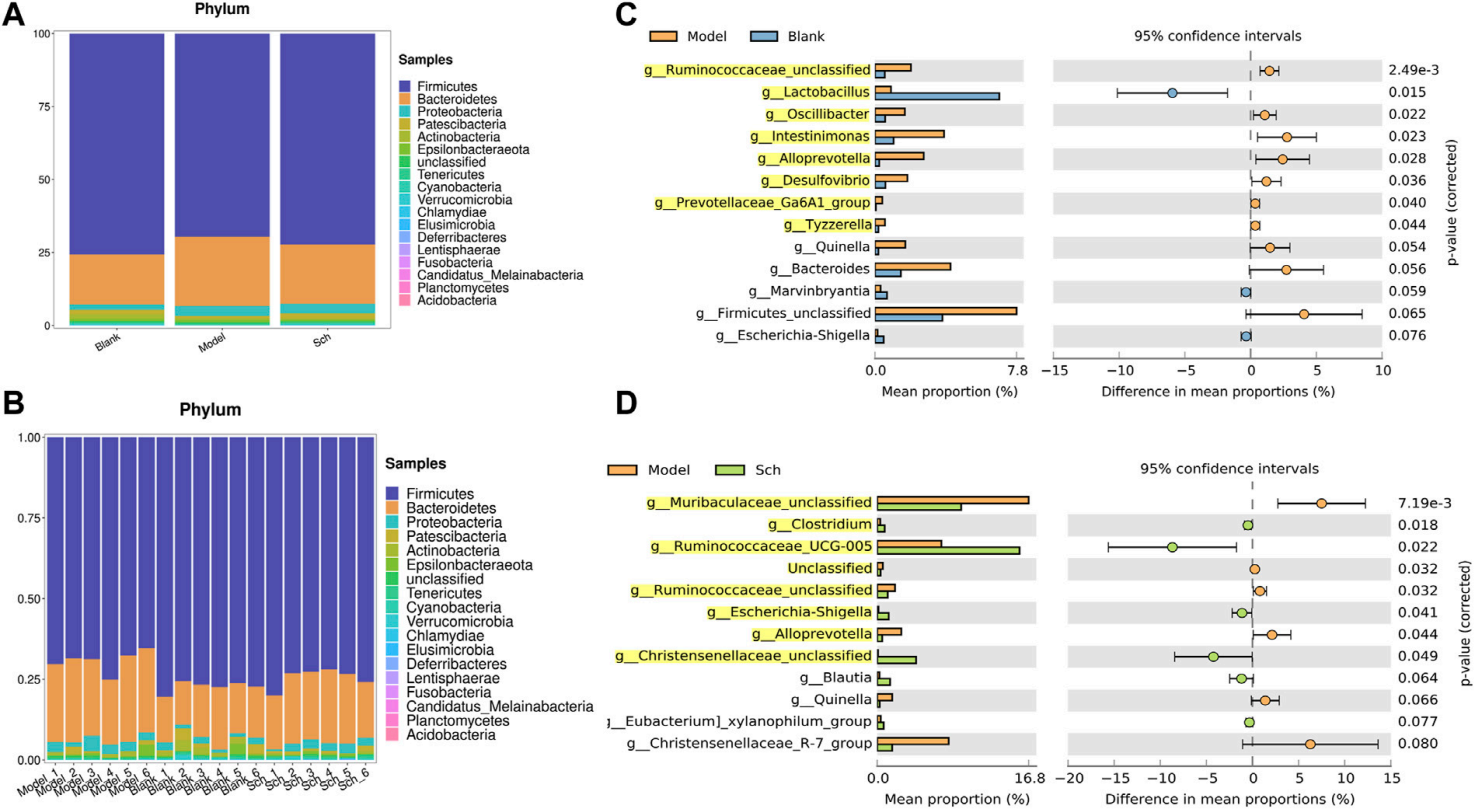
**(A,B)** Relative abundance of GM at the phylum level in the model group, blank group, and Sch group (%); **(C)** GM with significant differences between the genus level model group and the blank group; and **(D)** GM with significant differences between the genus level model group and the blank group. Analysis shows the relative abundance of microbial genus based on Welch’s test (*p* ≤ 0.05). The colored circles represent 95% confidence intervals calculated using Welch’s inverted method. GM with significant differences (*p* ≤ 0.05) is highlighted.

**FIGURE 9:**
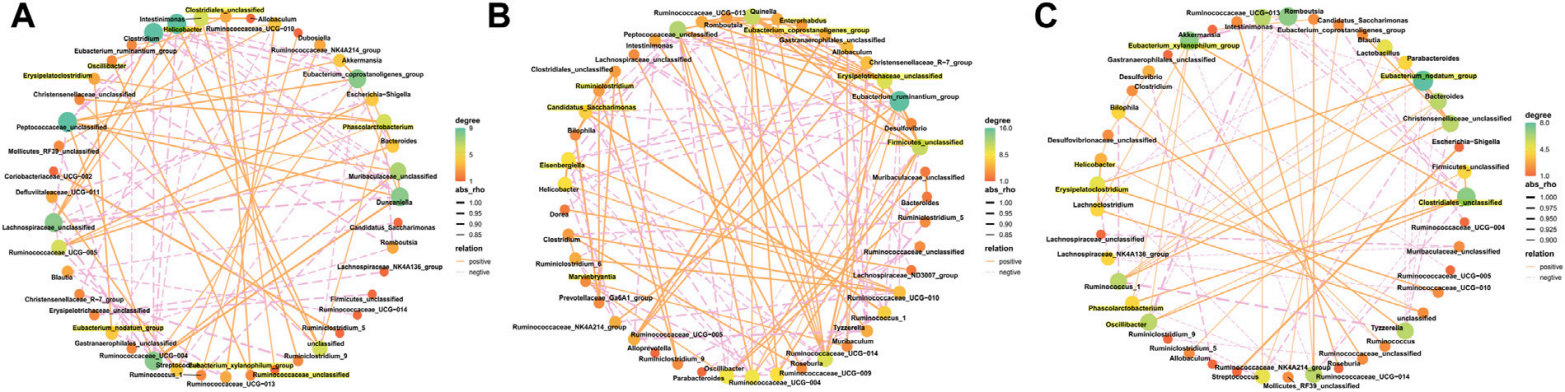
Correlated network of GM in the blank group **(A)**, model group **(B)**, and Sch group **(C)**. Significant strong Spearman correlations (*p* ≤ 0.05, | rho |> 0.5) among the GM are presented in the network. The highlighting means that bacteria are emphasized in the results section. Color and size of the dots represent the center degree. The solid orange line represents positive correlation; the dotted pink line represents negative correlation. The thickness of the line represents the level of correlation, and the thicker line represents the greater correlation.

The results from this analysis revealed that the relative abundance of firmicutes phylum in the Sch and blank groups increased, whereas, the relative abundance of the bacteroides phylum decreased (Figures 8A,B). The results from the top 50 relatively abundant bacterial community indicated that multiple microorganisms showed significant differences at the genus level (*p* ≤ 0.05) (Figures 8C,D). The content of bacteroides, escherichia-Shigella, ruminococcaceae_unclassified, oscillibacter, and intestinimonas in the model group tyzzerella, alloprevotella, firmicutes_unclassified, and quinella increased as compared to the blank group. However, the content of *lactobacillus* and muribaculaceae_unclassified decreased. However, the *lactobacillus* content, ruminococcaceae_UCG-005, muribaculaceae_unclassified, *clostridium*, and christensenellaceae_unclassified increased in the Sch group as compared to the model group, while the content of escherichia-Shigella, enterorhabdus, bacteroides, ruminococcaceae_unclassified, and alloprevotella decreased in the same group. To further study the changes being occurring in the GM at the genus level, the top 50 bacteria with the highest relative abundance in all the three groups were selected for Spearman’s correlation analysis (Figures 9A-C) (*p* ≤ 0.05, rho ±0.5). The correlation results showed that the mutual symbiosis relationships among the bacteria in all the three groups has changed significantly. AD severely interfered with the symbiosis of GM in the diseased rats. Schisandrin showed improvement in the imbalance of symbiosis, for example, in the AD model group, eisenbergiella, enterorhabdus, firmicutes_unclassified, marvinbryantia, quinella, candidatus_saccharimonas, and the other GMs have established new symbiotic relationships. The newly established symbiotic relationship of these GMs have disappeared right after the schisandrin treatment. Some GMs, which were also treated with schisandrin, have re-established the same correlation as the blank group compared to the model group. For example, clostridiales_unclassified and oscillibacter and eubacterium_xylanophilum_group, erysipelatoclostridium and eubacterium_nodatum_group have re-established a positive correlation. On the other hand, phascolarctobacterium and romboutsia have established a negative correlation, indicating that schisandrin had strengthen the symbiotic relationships between them. The changes occurred in the correlation between GM indicate that AD might have interfered with the symbiotic relationship between the GM. Schisandrin had improved the imbalance of this relationship to a certain extent, thereby suggesting that this balanced relationship is closely related to the development mechanism of schisandrin to play a protective effect on the rats with AD.

### 3.5 Predictive metagenomics analysis of the GM

**FIGURE 10:**
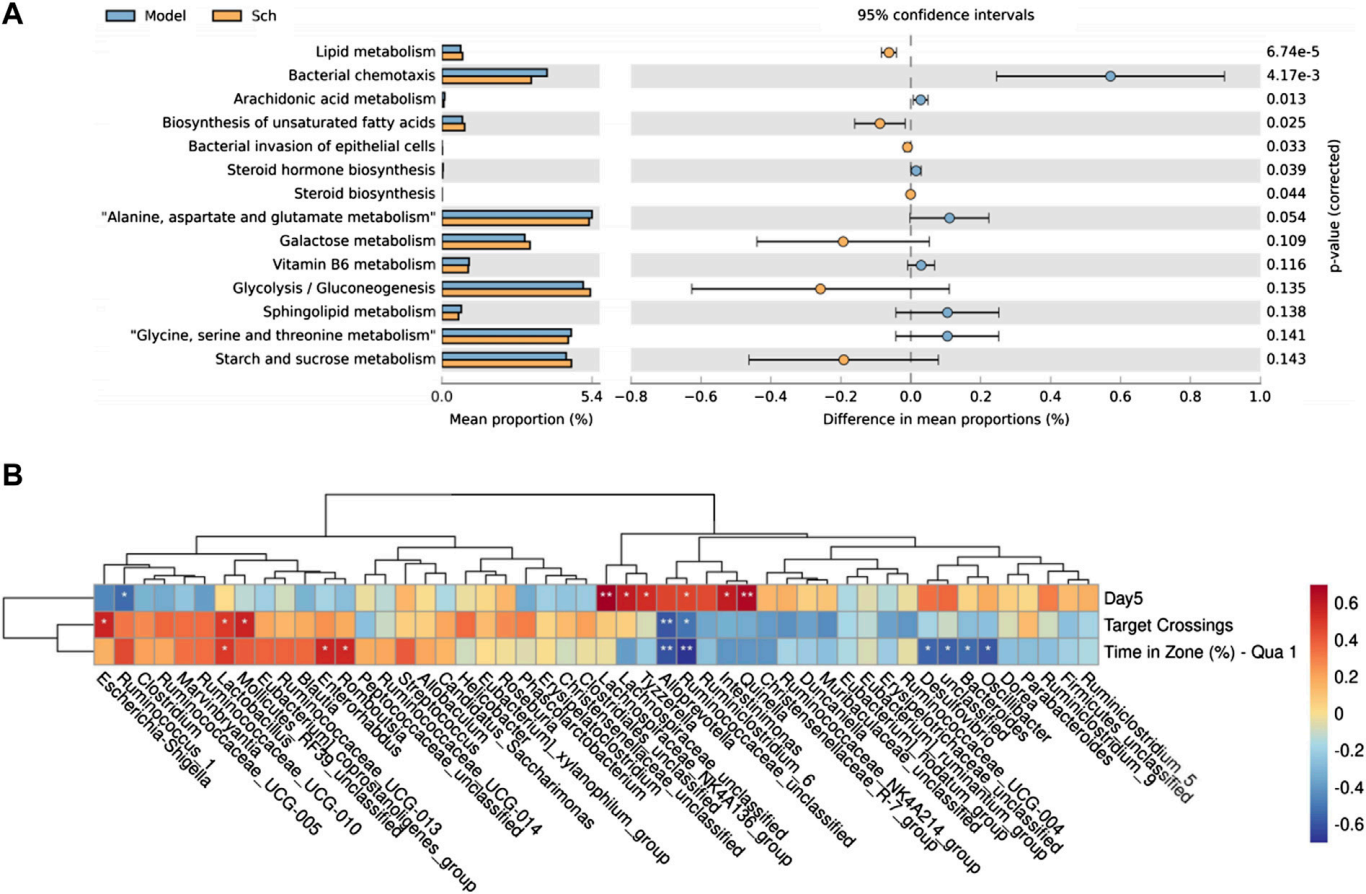
**(A)** PICRUSt2 analysis based KEGG database function prediction in the model group and Sch group; **(B)** Spearman’s correlation clustering heatmap between the GM and cognitive impairment (*p ≤ 0.05, **p ≤ 0.01). The color trend toward red represents positive correlation, and the color trend toward blue represents negative correlation. Day 5 is the escape latency of the rats on the fifth day of the space exploration experiment, target crossing is the number of times that the rats crosses the escape platform, and time in zone (%)-Qua one is time in first quadrant in spatial exploration test.

Based on PICRUSt2, we observed the potential functional changes in the GM of rats from the KEGG database (Figure 10A). A number of metabolic pathways and functions of the GM in the model and Sch groups were significantly enriched, including lipid metabolism, steroid hormone biosynthesis, arachidonic acid metabolism, biosynthesis of unsaturated fatty acids, bacterial invasion of epithelial cells, and steroid biosynthesis.

### 3.6 Relationship between the GM and cognitive impairment of rats with AD

To reveal the pathological significance of the changes being occurring in the GM of rats, top 50 relatively abundant GMs were selected to analyze the correlation between their abundance and behavior (Figure 10B). The correlation results revealed that among the tract bacteria, escherichia-Shigella, rumenococcus_1, *lactobacillus*, *lactobacillus*_RF39_unclassified, *enterobacter*, rumbutzia, lacetospiraceae_NK4A136_group, tyzzerella, lactococcus, *enterobacter*, quininella, desulfovibrio, and bacteroides found to have a significant correlation with the behavior, *lactobacillus* and bacteroides were the two most common abundant bacteria among them, thereby suggesting their critical role in the mechanism of schisandrin treatment.

### 3.8 Potential relationships between the host metabolites and GM

**FIGURE 11:**
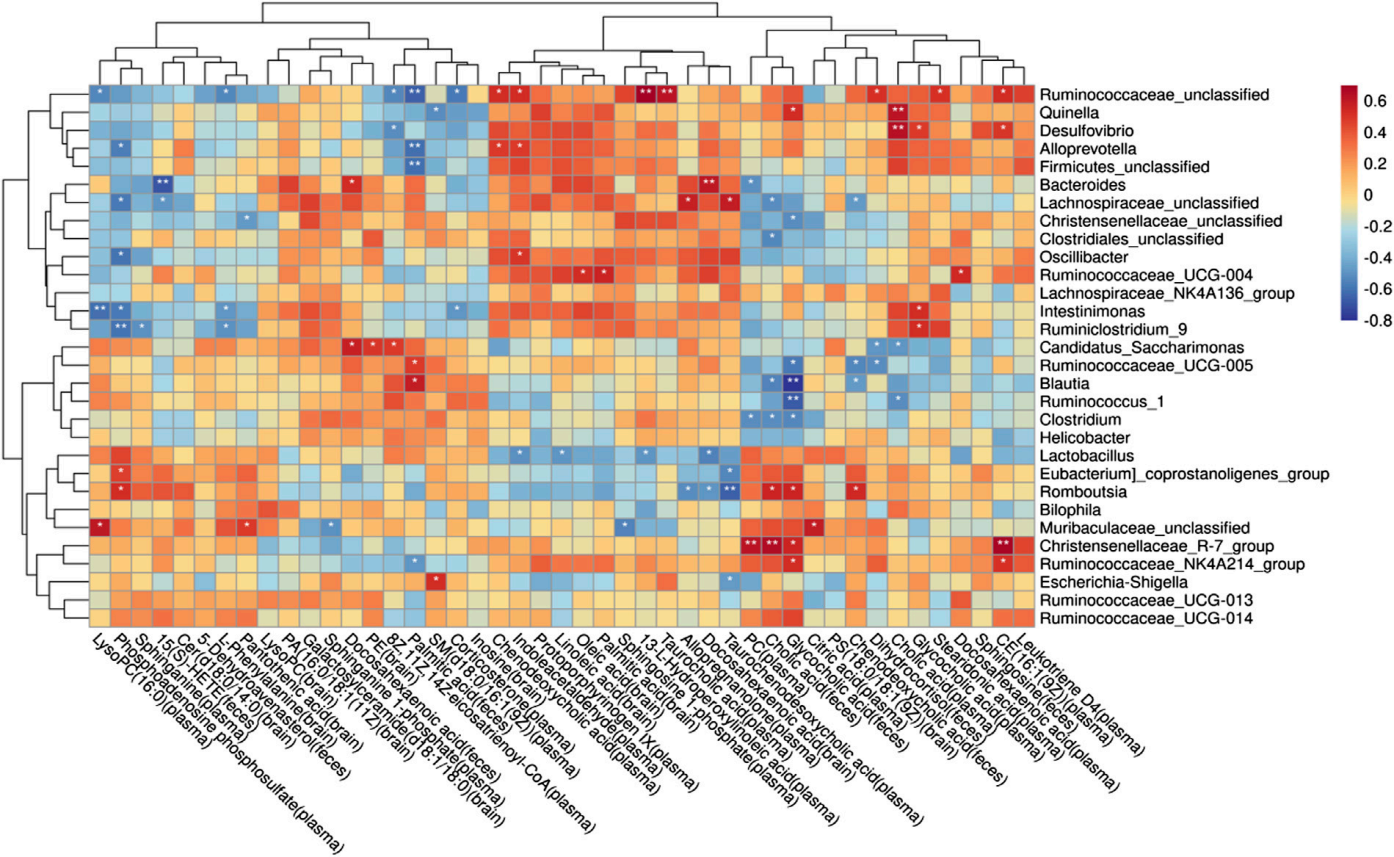
Potential relationship between metabolites and gut microbiota in rats with AD after schisandrin treatment.

To understand the mechanism of metabolic disorder in the GM caused by the schisandrin in improving the symptoms of diseased rats, the potential differential metabolites enriched in the brain, feces, and plasma sample pathways were selected. Spearman’s correlation coefficient between the metabolites and the top 50 abundant GMs was calculated (Figure 11). The results indicate that most of the GM had a significant correlation with the metabolites in the body. In the plasma samples, citric acid, phenylpyruvic acid, tetradecanoyl-CoA, and 3-Oxo-OPC6-CoA were found positively correlated with christensenellaceae_R-7_group, whereas, christensenellaceae_unclassified, lachnospiraceae_unclassified, oscillibacter, intestinimonas, ruminiclostribacter, intestinimonas, ruminiclostriovic acid, tetradecanoyl-Phenylco Oxo-OPC6-CoA, corticosterone, PC (14:0/22:4 (7Z, 10Z, 13Z, 16Z)), arachidonate, 8Z, 11Z, and 14Z-eicosatrienoyl-CoA were found negatively correlated. In the brain, arachidonate, docosahexaenoic acid, oleic acid, linoleic acid, glycocholic acid, galactosylceramide (d18:1/18:0) were associated with the bacteroides, christensenellaceae_unclassified, phascolarctobacterium, parabacteroides, lachnospiraceae_unclassified, and oscillibacterium, whereas, enterbacillibacteria, edus _ruminantium_group, and peptococcaceae_unclassified were found negatively correlated. In the feces samples, alloprevotella and ruminiclostridium_5 were negatively correlated with myristic acid and palmitic acid, whereas, hydroxylidocaine was positively correlated with LysoPC (P-18:0), dihydrocortisol, and glycocholic acid.

## 4 Discussion

**FIGURE 12:**
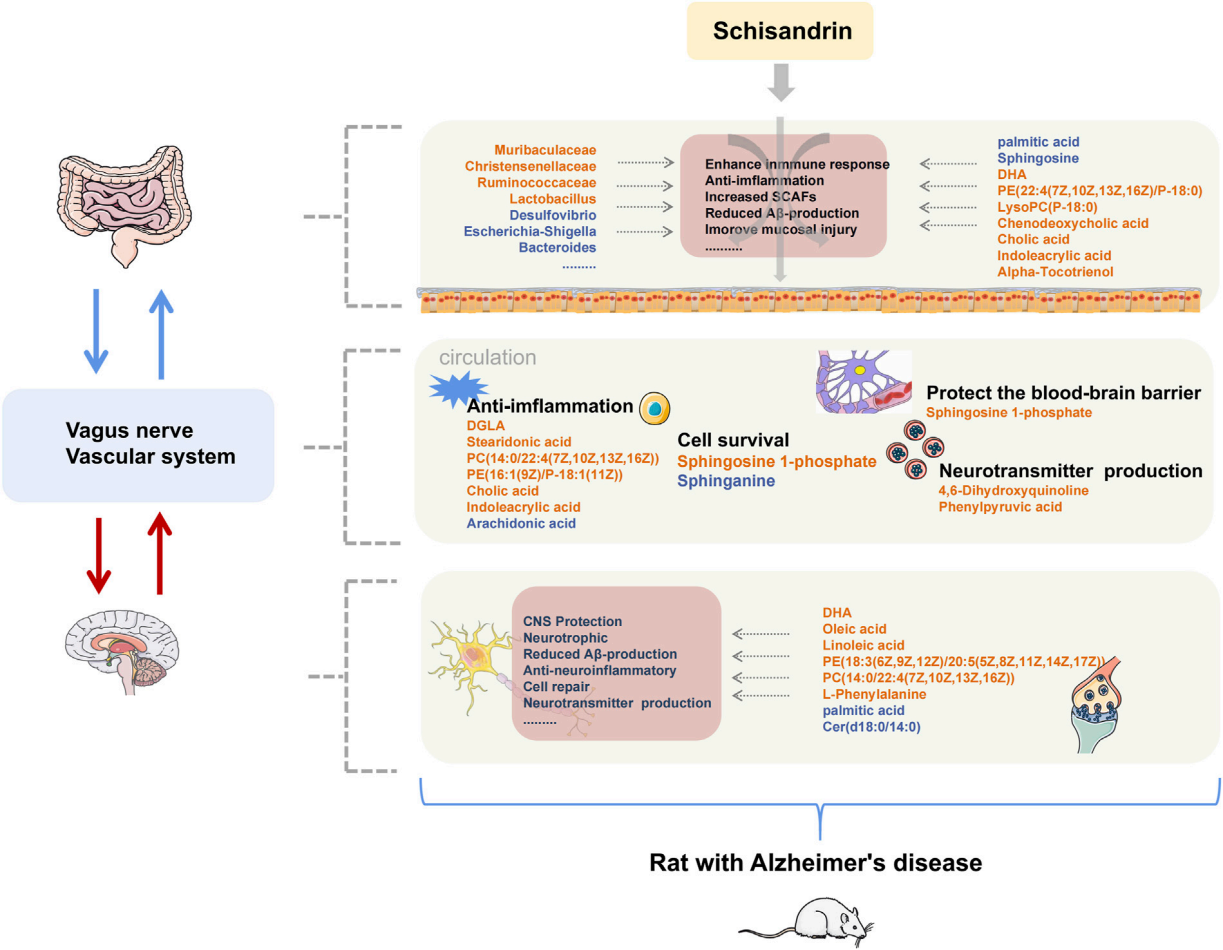
Summary of changes in rats with AD after treatment with schisandrin. The font color of endogenous metabolites and GM represent the content level and abundance, orange represents an increase, and blue represents a decrease.

AD is a neurodegenerative disease with insidious onset and reportedly has an excellent therapeutic effect on AD. Schisandrin can inhibit neuroinflammation, Aβ deposition, and helps improving the cognitive impairment in AD. In this study, we have tried to comprehensively explore the pathogenesis of AD and the protective effect of schisandrin on rats with AD through the metabolomics analysis of brain, plasma, and fecal nontargeted combined with 16S rDNA gene sequencing technology systematically from the brain–gut axis (Figure 12). In this regard, an AD rat model was developed by injecting D-gal intraperitoneally, orally administering AlCl_3_, and bilaterally injecting Aβ_25-35_ intraventricularly to induce the AD. AlCl_3_ can induce the rats to produce progressive neurodegeneration similar to that observed in AD (Ulusoy et al., 2015), D-gal is an aging agent that causes cognitive impairment (Cao et al., 2019), and Aβ deposition is the main pathology of AD (Gupta, Sil et al., 2018). Collectively, these three methodologies have a synergistic effect on the establishment of AD rat models (Chiroma et al., 2018; McDonald et al., 2021).

Identified metabolites with large fluctuations in the metabolic levels were mainly enriched in unsaturated fatty acid biosynthesis, sphingolipid metabolism, primary bile acid biosynthesis, glycerophospholipid metabolism, and tryptophan metabolism, which were also significantly correlated with the identified GM. Several studies have shown that multiple GMs were associated with the occurrence and development of the aforementioned metabolic pathways. The prediction of GMs pathway further confirmed that the aforementioned metabolic pathways were directly involved in the anti-AD mechanism of schisandrin. Therefore, we focused on the stated metabolic pathways to discuss the mode of action of schisandrin.

The results from the MWM test and H&E staining suggest that schisandrin is directly involved in the improvement of cognitive impairment caused by AD, and could also reduce the loss of hippocampal neurons in rats. The Firmicutes/Bacteroidetes (F/B) ratio of the model group was also decreased, indicating an imbalance in the GM. At the genus level, some microbes showed significant differences among all the three study groups. Muribaculaceae family might have played an essential role in degrading the mucosal layer (Evans et al., 2014; Rooks et al., 2014). Similarly, desulfovibrio can cause a significant reduction in the short-chain fatty acids (SCFAs) levels (Sawin et al., 2015), because the enterobacteriaceae and desulfovibrio bacteria are the main lipopolysaccharide (LPS) endotoxin-producing bacteria (Zhao 2013). Schisandrin inhibits desulfovibrio bacteria and helps in protecting the integrity of intestinal endothelium. Based on the inhibitory effect of schisandrin on desulfovibrio, it can be deduced that it has profound effects on the treatment of AD. Similarly, christensenellaceae is one of the five taxa considered as a sign of healthy intestines. In our case, we have observed that the christensenellaceae content in the Sch group was increased significantly, thereby indicating that schisandrin can restore the intestinal environment to a healthy state by multiplying the content of christensenellaceae in the gut. Furthermore, ruminococcaceae is responsible for the fermentation of dietary fiber and other plant components, and schisandrin has caused a significant increase in the ruminococcaceae content, thus indicating that it can metabolize to produce more SCFAs. Escherichia–shigella is positively correlated with the expression of cytokine and is a typical intestinal pathogen (Chatterjee et al., 2018). We have observed a significant decrease in the escherichia-Shigella content after treating with schisandrin. Taken together, the GM function in diseased rats treated with schisandrin is found to be involved in the bacterial chemotaxis, arachidonic acid metabolism, biosynthesis of unsaturated fatty acids, and bacterial invasion of epithelial cells, which further confirms the improvement of gut inflammation caused by the GM invasion.

It is well known that the supplementation of docosahexaenoic acid (DHA) and eicosapentaenoic acid (EPA) can improve the cognitive impairment in AD patients and these can also reduce the neuroinflammation. Elevated palmitic acid levels are related to the cognitive impairment in humans. Oleic acid improves amyloidosis in AD cells and has neurotrophic effects. Linoleic acid can reverse the inflammatory response of microglia and help in controlling the inflammatory cell response in the brain by targeting the myeloid cells in the gut. Serum dihomogamma-linolenic acid (DGLA), with its anti-inflammatory effects, is associated with the cognitive functions and mild cognitive impairment at much reduced levels in patients with coronary artery disease (Ishihara et al., 2020). Furthermore, stearidonic acid can increase the concentration of EPA in the plasma cells, and arachidonic acid on the other hand is directly or indirectly converted to eicosanoid, thereby leading to cause cell impairment and mediates inflammation. In the present study, schisandrin treated rats had higher levels of DHA, oleic acid, and linoleic acid in the brain, whereas, the palmitic acid was recorded at lower levels. Similarly, in the plasma, Sch group had higher levels of DGLA, stearidonic acid, and lower levels of arachidonate. Regarding the feces, palmitic acid content was reduced and the DHA content was increased. Collectively, our results suggest that schisandrin can improve AD’s inflammatory state, can maintain the metabolic balance, and can impart nutritional effect on the brain.

According to previous studies, the levels of phosphatidylcholine (PC), phosphatidylethanolamine (PE), and phosphatidylinositol (PI) in the neural membranes of different regions of AD patients were reduced as compared to the age-matched control human brains (Söderberg et al., 1991). In this study, the glycerophospholipid metabolic pathways in the brain, plasma, and feces were found disordered, and through the regulatory effect of schisandrin, the metabolic level gradually returned to normal.

Sphingolipid (SL) metabolism is related to the level of amyloid β in the cerebrospinal fluid of AD (Fonteh et al., 2015). Also, the GM of phylum bacteroides can produce SL. Through the prediction analysis of the GM function, it was observed that GM participates in the process of sphingolipid metabolism, and after production sphingolipids enter the host metabolic pathway to modulate the ceramide levels (Johnson et al., 2020). The interaction of sphingolipids and bacteria in the gut stimulates the activity of iNKT cells through CD1b, thereby exerting an immunomodulatory effect (An et al., 2014). In the nervous system, sphingosine-1-phosphate (S1P) can influence the blood–brain barrier when its level decreases in the blood (Vutukuri et al., 2018). In this study, the AD model group showed significant changes in the SL metabolism pathway, such as the decrease in S1P content in the plasma and the decrease in Galactosylceramide (d18:1/18:0) content in the brain. The metabolic level was reversed after schisandrin treatment, suggesting that it can protect diseased rats by changing the SL metabolism level and by improving the influence of microorganisms on SL metabolism.

Cholic acid and alpha-muricholic acid can inhibit the proliferation of *Escherichia coli*, *streptococcus*, and other harmful bacteria (Watanabe et al., 2017). Previous findings have elaborated that a decrease in the blood cholic acid is related to the reduction of the overall glucose metabolism in the brain, and the lower cholic acid content and higher chenodeoxycholic acid is associated with more severe brain atrophy (Nho et al., 2019). Similar to previous studies, the cholic acid content in the feces of diseased rats was reduced in our case, and it was adjusted after schisandrin treatment. The harmful bacteria, romboutsia and enterorhabdus, showed a significant negative correlation with cholic acid in feces, which further strengthens that excessive cholic acid might be involved in reducing some harmful bacteria. Furthermore, in the plasma samples, the cholic acid content in the blank and Sch groups was increased, thereby indicating that schisandrin can play a protective role by regulating the bile acid biosynthesis pathway in rats.

Tryptophan participates in the kynurenine pathway, and its metabolites can affect the central nervous system (CNS) through the metabolic pathways. In the model group, the content of the aforementioned metabolites was abnormal, and it was improved after schisandrin treatment was applied. Tryptophan is the only amino acid with an indole structure. Indoleacrylic acid transformed by the GM can promote mucosal homeostasis, and protect mice from bacterial infections, thereby reduce the gut inflammation (Wlodarska et al., 2017). Indoleacrylic acid has anti-inflammatory and antioxidant effect in human peripheral blood mononuclear cells activated by LPS by reducing interleukin (IL)-6 and IL-1β and activating the NRF2-ARE pathway (Joshi and Johnson 2012). In our case, the indoleacrylic acid content in the feces of rats was decreased in the model group. After schisandrin treatment, the indoleacrylic acid content was increased, thus suggesting that schisandrin can protect the gut–blood barrier through anti-inflammatory effect in protecting rats with AD.

## 5 Conclusion

Although the therapeutic effect of schisandrin had been confirmed in earlier studies, the underlying mechanism to improve the symptoms in rats diagnosed with AD was still unclear. In this context, our study provides convincing results about the mode of action of schisandrin. The metabolomics studies of the plasma, feces, and brain showed that schisandrin had reversed the imbalance of the metabolic level of the brain–gut axis in the diseased rats, and furthermore, it was also involved in the multiple metabolic pathways in AD. Moreover, 16S rDNA gene sequencing results revealed that schisandrin improved the GM imbalance and gut–blood barrier damage caused by gut inflammation in rats. Finally, Spearman’s correlation analysis showed that the changes in intestinal microbes were significantly related to the overall metabolic level in the body. The overall data underline that although schisandrin has low oral bioavailability, it can still improve the metabolic imbalance of rats with AD by strengthening the gut microenvironment.

## Data Availability

Original datasets are available in a publicly accessible repository: The original contributions presented in the study are publicly available. This data can be found here: [SUB11896391].
