# Supplementary material for: Schisandrin alleviates the cognitive impairment in rats with Alzheimer’s disease by altering the gut microbiota composition to modulate the levels of endogenous metabolites in the plasma, brain, and feces
Source: Front Pharmacol. 2022 Sep 12;13:888726. doi: 10.3389/fphar.2022.888726 (PMC9514097; doi:10.3389/fphar.2022.888726)
Supplement: Supplementary file 1 [file DataSheet1.docx]

**Supplementary Figures**

**
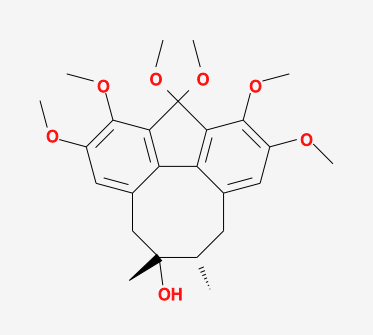
**

Supplementary Figure 1: The chemical structure of Schisandrin.


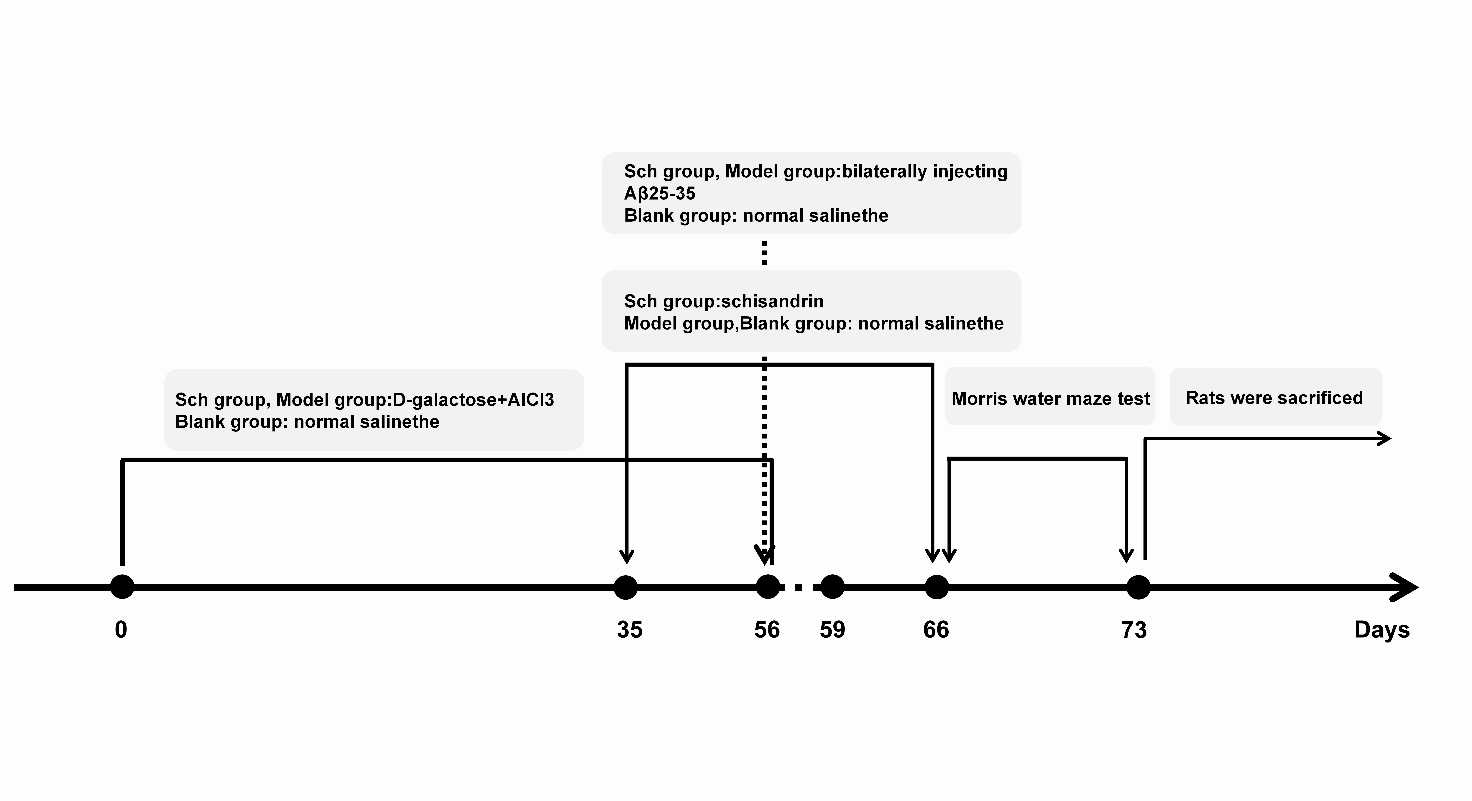
Supplementary Figure 2: The experimental design time was arranged.


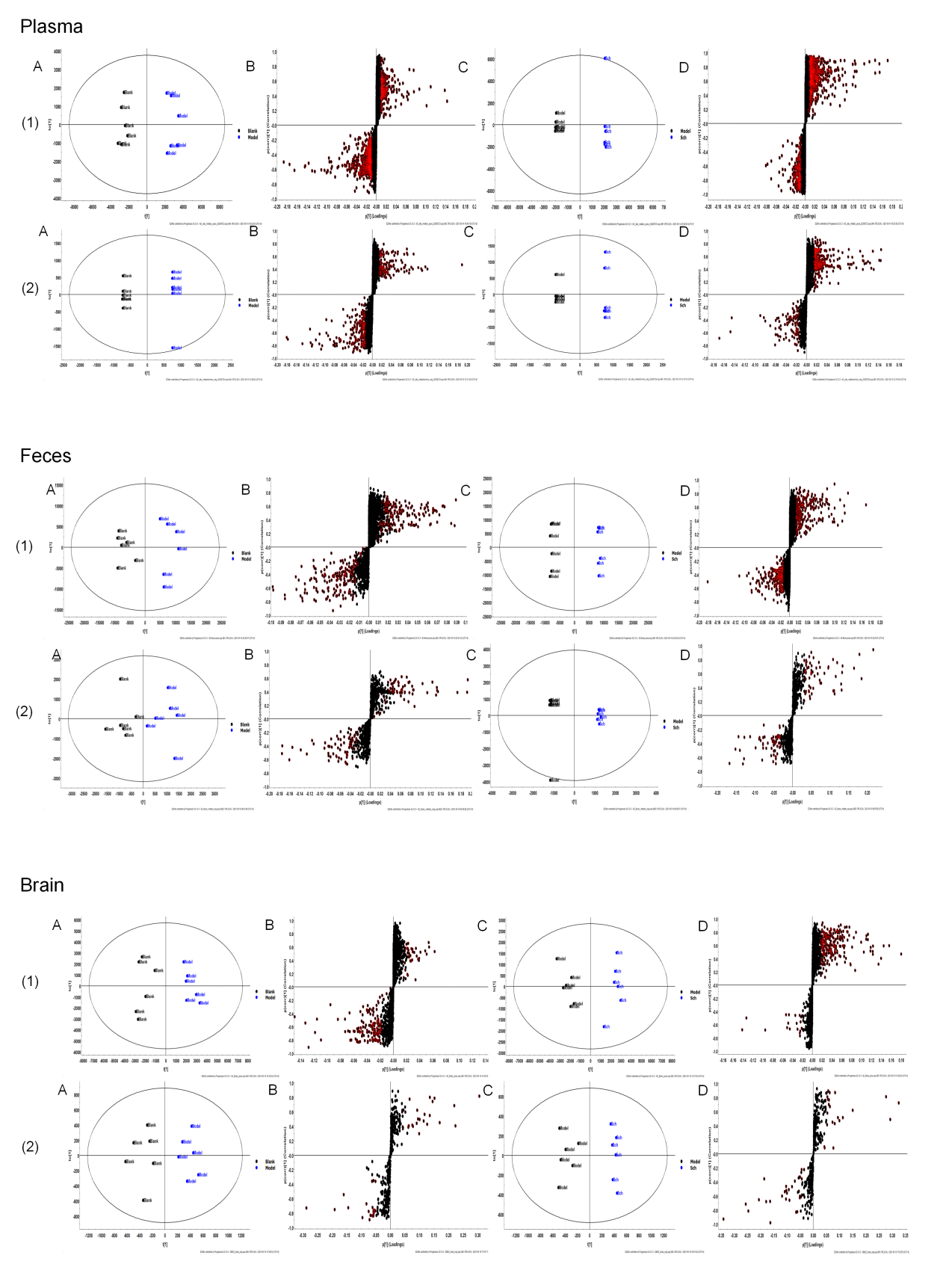


Supplementary Figure 3: Multivariate statistical analysis of plasma 、feces and brain metabolomics. In the positive ion mode (1), (A) OPLS-DA score plot of blank group and model group; (B)S-plot of blank group and model group; (C) OPLS-DA score plot of model group and Sch group; (D) S-plot of model group and Sch group; In the negative ion mode (2): (A) OPLS-DA score plot of blank group and model group; (B)S-plot of blank group and model group; (C) OPLS-DA score plot of model group and Sch group; (D) S-plot of model group and Sch group.
